# Two Different Secondary Metabolism Gene Clusters Occupied the Same Ancestral Locus in Fungal Dermatophytes of the Arthrodermataceae

**DOI:** 10.1371/journal.pone.0041903

**Published:** 2012-07-30

**Authors:** Han Zhang, Antonis Rokas, Jason C. Slot

**Affiliations:** Department of Biological Sciences, Vanderbilt University, Nashville, Tennessee, United States of America; Pacific Northwest National Laboratory, United States of America

## Abstract

**Background:**

Dermatophyte fungi of the family Arthrodermataceae (Eurotiomycetes) colonize keratinized tissue, such as skin, frequently causing superficial mycoses in humans and other mammals, reptiles, and birds. Competition with native microflora likely underlies the propensity of these dermatophytes to produce a diversity of antibiotics and compounds for scavenging iron, which is extremely scarce, as well as the presence of an unusually large number of putative secondary metabolism gene clusters, most of which contain non-ribosomal peptide synthetases (NRPS), in their genomes. To better understand the historical origins and diversification of NRPS-containing gene clusters we examined the evolution of a variable locus (VL) that exists in one of three alternative conformations among the genomes of seven dermatophyte species.

**Results:**

The first conformation of the VL (termed VLA) contains only 539 base pairs of sequence and lacks protein-coding genes, whereas the other two conformations (termed VLB and VLC) span 36 Kb and 27 Kb and contain 12 and 10 genes, respectively. Interestingly, both VLB and VLC appear to contain distinct secondary metabolism gene clusters; VLB contains a NRPS gene as well as four porphyrin metabolism genes never found to be physically linked in the genomes of 128 other fungal species, whereas VLC also contains a NRPS gene as well as several others typically found associated with secondary metabolism gene clusters. Phylogenetic evidence suggests that the VL locus was present in the ancestor of all seven species achieving its present distribution through subsequent differential losses or retentions of specific conformations.

**Conclusions:**

We propose that the existence of variable loci, similar to the one we studied, in fungal genomes could potentially explain the dramatic differences in secondary metabolic diversity between closely related species of filamentous fungi, and contribute to host adaptation and the generation of metabolic diversity.

## Introduction

Dermatophyte fungi of the family Arthrodermataceae (Eurotiomycetes), including *Trichophyton* and *Microsporum* (teleomorph *Arthroderma*) species, colonize keratinized tissue of humans and other vertebrates and are the most common cause of superficial mycoses [1]. Colonization of the animal epidermis environment is challenging due to nutrient scarcity and competition with the native microflora [2]. For example, bacterial inhabitants of human skin lower surface pH, secrete defense compounds and compete for scarce nutrients such as iron [2]. To overcome these challenges, dermatophytes have specialized to produce siderophores, such as ferrocrocin and ferrochrome C, for efficient iron scavenging [3], as well as antibiotic compounds that are active against skin bacteria [4]. Penicillin resistance can evolve in the bacterium *Staphylococcus aureus* during *Trichophyton* spp. infections [5], underscoring the evolutionary arms race between native and invading skin microbes.

In dermatophytes, and more generally in filamentous fungi, the genes involved in the production of antibiotics, siderophores, and other secondary metabolism compounds, are often physically linked or clustered in the genome. Recent work suggests that such gene clusters can facilitate rapid genome remodeling, adaptation to new niches, and acquisition of novel functions through gain of entire pathways via horizontal gene transfer, as well as through their wholesale loss [6]–[12]. Compared to dermatophytes from other lineages of fungi and bacteria, the Arthrodermataceae possess a large number of secondary metabolism gene clusters thought to be involved in host specificity and pathogenicity [1]. The majority of these gene clusters contain non-ribosomal peptide synthetase (NRPS) genes, which are generally known to produce compounds such as beta-lactams, statins, and siderophores [13], [14]. The very large number of secondary metabolism gene clusters in Arthrodermataceae dermatophytes, coupled with the knowledge that their distribution varies substantially among closely related species [1], suggests that a highly diverse set of secondary metabolism compounds are involved in the colonization of epidermis.

Although NRPS gene cluster diversity is well documented, and the evolutionary history of NRPS genes well characterized [15], relatively little is known about the historical origins and diversification of NRPS-containing gene clusters and of secondary metabolism clusters in general. To address this question we examined a locus (termed variable locus or VL) otherwise highly conserved in microsynteny that exists in one of three alternative conformations among the seven fully sequenced dermatophyte genomes. The three conformations contain distinct sets of zero (VL conformation A, or VLA), twelve (conformation VLB), and ten (conformation VLC) genes in *Microsporum canis, M. gypseum*, and *Trichophyton* spp., respectively. Interestingly, we found that the VLB and VLC conformations contain distinct and unique putative NRPS secondary metabolism gene clusters. Phylogenetic evidence suggests that that the ancestor of all seven species was either polymorphic for VLB and VLC or contained them both and subsequent differential loss or retention of alternative conformations resulted in their present distribution. We propose that the existence of such variable loci could explain the dramatic differences in secondary metabolism pathway content between closely related species of filamentous fungi, and contribute to host adaptation and the generation of metabolic diversity.

## Methods

### Analyses of Fungal Gene Order

The variable locus (VL) was identified in *Microsporum gypseum* during a search for chromosomal clusters of genes that correspond to two or more enzymes of the porphyrin and chlorophyll metabolism pathway (KEGG #00860) [16]. We used NCBI BLASTP [17] searches against a local database of 128 fungal genomes (Table S1), and inferred homologous enzyme gene families using the OrthoMCL software, version 1.4 [18]. We considered individual members from different gene families to be physically clustered in the genome based on two criteria: (a) six or fewer intervening genes separated them, a distance chosen empirically from distributions observed in previous analyses [6]–[8], and evaluated for significance by pathway randomization (data not shown), and (b) the resultant gene cluster contained at least one pair of genes that are adjacent in a metabolic pathway in order to enrich for biochemically meaningful clustering. We then inferred the sequence content of the same locus in six other dermatophyte genomes that are publicly available (*Microsporum canis*, as well as *Arthroderma benhemiae*, *Trichophyton verrucosum*, *T. rubrum*, *T. tonsurans*, and *T. equinum*, which we collectively refer to as *Trichophyton* spp.) by identifying syntenic stretches of greater than four putative homologs of the *M. gypseum* genes. We confirmed synteny by whole scaffold nucleotide alignments using the progressive algorithm with a HOXD scoring matrix in the Mauve software, version 2.3.1 [19]. Finally, we functionally characterized the sequence content of the locus in the other six closely related dermatophyte genomes by comparison to annotated proteins in GenBank, and we inferred functional domains using the NCBI conserved domain database [20].

### Phylogenetic Analysis

For each gene in the VL as well as for the first six genes on the 5′ flank of the VL and of ten genes on the 3′ flank, we retrieved protein sequences from the GenBank *nr* database and a local database of 128 fungal and 1,130 bacterial complete genomes using the BLASTP algorithm. We retained hits that were >45% similar at the amino acid level and between 50% and 150% the length of the query. We aligned amino acid sequences with the MAFFT software, version 6.847, under default settings [21]. Phylogenetic analysis of each gene set was performed in two steps. First, we performed a maximum parsimony (MP) analysis using the PAUP* software, version 4.0b10 [22] on the sequence alignment following the removal of alignment columns containing greater than 40% gaps using the TrimAL software, version 3 [23]. After examining the robustness of the inferred tree topology using 100 bootstrap replicates, we then used the MP majority rule bootstrap consensus tree as a guide to reduce the alignment to contain only the branches most closely related to sequences from the VL. We then realigned this reduced set of protein sequences, removed alignment columns as described above, and performed maximum likelihood analysis using the RAxML software, version 7.2.6 [24], under the PROTGAMMAJTT model of amino acid substitution. We examined tree topological robustness with 100 bootstrap replicates. We also run analyses under other commonly used models of amino acid substitution with no significant changes in our results (data not shown). We performed comparative topology analyses using the Shimodaira-Hasegawa test [25], as implemented in RAxML, and parsimonious reconstruction of gene duplication and loss events in Notung 2.6 [26].

## Results

### Three Alternative Conformations Exist in the Same Locus in Dermatophytes

We detected a unique cluster of four genes involved in porphyrin metabolism in the genome of *Microsporum gypseum*. Examination of the genomic region containing this gene cluster in the genomes of six related dermatophyte species showed that the region differed greatly in both size and gene content in the other six genomes, even though it was nested within an otherwise highly conserved 500 Kb segment in all seven genomes (Figure S1). We designate this variable genomic region across the seven dermatophyte genomes as the variable locus (VL).

**Figure 1:**
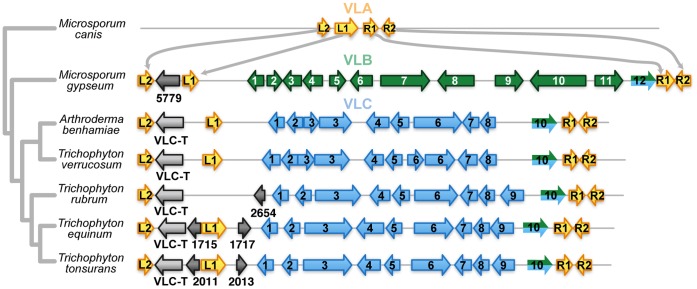
Synteny and the three distinct conformations of the variable locus. The consensus species phylogeny of *Microsporum canis*, *Microsporum gypseum*, and *Trichophyton* spp. is shown on the left. The gene contents of the variable locus of each species are shown to the right of the species name. The yellow arrows represent the genes flanking the variable locus. Arrows with the same label (i.e., L1) represent homologous genes. The green arrows represent the genes in the variable locus in *Microsporum gypseum*. These genes are flanked by conserved genes L1, L2, R1, and R2. The blue arrows represent the genes found in the variable locus in the *Trichophyton* spp. The conserved genes, marked in yellow, also flank the *Trichophyton* spp. genes in the variable locus. However, in *Microsporum canis*, the conserved flanking genes (marked by yellow) are directly connected. The grey arrows represent lineage-specific intervening genes. Both the assemblages in *Trichophyton* spp. and *M. gypseum* share the flanking gene VLB-12/VLC-10 (marked half-green, half-blue). These arrows are drawn to scale.

The VL exists in three distinct conformations (Figure 1). In *M. canis*, the VL locus is 539 base pairs long and apparently lacks any protein-coding sequences (VLA conformation). In *M. gypseum*, the VL is 35.89 Kb and contains 12 protein-coding genes (VLB conformation; contains genes VLB-1 through VLB-12). In *Trichophyton* spp. the VL is 26.78 Kb and contains 10 protein-coding genes (VLC conformation; contains genes VLC-1 through VLC-10). Both the 5′ and 3′ flanks of the VL contain several stretches of contiguous homologous genes that are conserved in synteny across the seven genomes, including VL-L1 and VL-L2, the first and second genes immediately adjacent to the 5′ flank of VL, and VL-R1 and VL-R2, the first and second genes immediately adjacent to the 5′ flank of VL.

### Two of the Three Variable Locus Conformations Contain NRPS Gene Clusters

**Figure 2:**
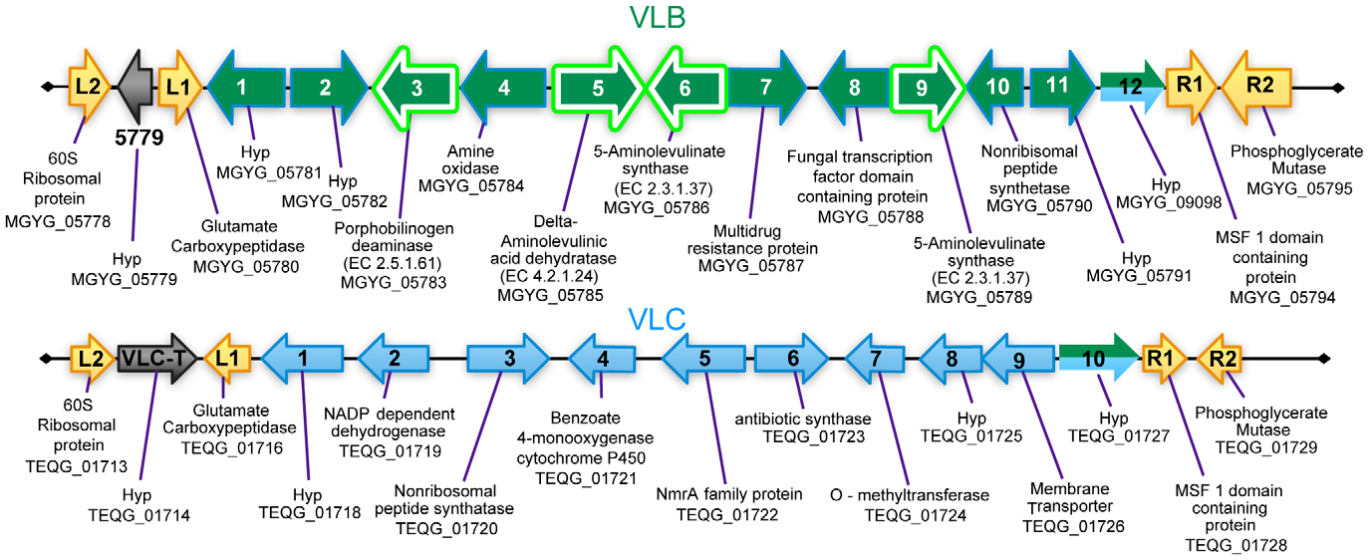
Functional annotation of genes in the two putative NRPS clusters . Two assemblages of genes found in the VL in *M. gypseum* and in *Trichophyton* spp. are displayed. The functional annotation of each gene is shown. The green arrows represent proteins in the VLB locus state (in *M. gypseum*), predicted to be involved in secondary metabolite synthesis and transport. Porphyrin metabolism genes are outlined in white. The blue arrows represent a consensus of the proteins in the VLC locus state (in *Trichophyton* spp.). These genes are predicted to produce and transport another type of undetermined secondary metabolite. The yellow arrows represent conserved proteins flanking the VL. The protein shared between the VLB assemblage in *M. gypseum* and VLC assemblage in *Trichophyton* spp. is shown half-green, half-blue. Hyp  =  hypothetical protein. Figure is not drawn to scale.

To better understand the genetic structure of the VL, we systematically annotated the protein-coding sequences in the two conformations VLB and VLC and found that both contained distinct putative NRPS gene clusters (Figure 2). In the VLB, aside from the NRPS gene (VLB-10), which contains an adenylation domain, a phosphopantetheine attachment site, condensation domain, and an acyl-protein synthetase, four other VLB genes (VLB-3, VLB-5, VLB-6, and VLB-9) are homologous to genes encoding for enzymes required to produce porphyrins from a glycine substrate (KEGG #00860). Two other genes in the cluster are also commonly associated with secondary metabolism gene clusters. Specifically, VLB-7 encodes a multidrug resistance pump containing a P-loop NTPase superfamily motif known to be present in ATP binding cassette proteins, whereas VLB-8 encodes a transcription factor related protein with a GAL4-like Zn2Cys6 binuclear cluster DNA-binding domain. Finally, the VLB-1, VLB-2, VLB-11, and VLB-12 genes encode for unknown or hypothetical proteins.

The VLC gene cluster, in addition to the NRPS gene (VLC-3), which contains an adenylation domain, a condensation domain, and a phosphopantetheine attachment site, also contains several other genes that are hallmarks for secondary metabolism-associated functions. Specifically, the VLC-5 gene encodes an NmrA family transcription regulator, VLC-6 an antibiotic synthase that contains a thioester reductase, adenylation domains, and a phosphopantetheine attachment site, whereas VLC-9 encodes a MFS membrane transporter. Additionally, VLC-2 encodes an NADP dependent dehydrogenase (VLC-2) with a FabG domain (COG1028), VLC-4 a cytochrome P450 enzyme with an Amine Oxidase domain (COG1231) that is associated with deamination of toxins and other compounds, and VLC-7 an O-methyltransferase. Finally, the VLC-1, VLC-8, and VLC-10 genes encode unknown or hypothetical proteins.

### Paralogs of VLB and VLC Genes are Differentially Distributed in Dermatophytes

To understand the evolutionary history of the genomic region containing the VL, we reconstructed the phylogenies of the first six genes on the 5′ flank of the VL and of ten genes on the 3′ flank. Our analyses show that fifteen of the sixteen genes are consistent with dermatophyte species relationships [27] and have not undergone dermatophyte-specific gene duplications. Only one gene on the 3′ flank, R9, was duplicated before the divergence of dermatophytes but is otherwise also consistent with species relationships (Figure S2AG).

**Figure 3:**
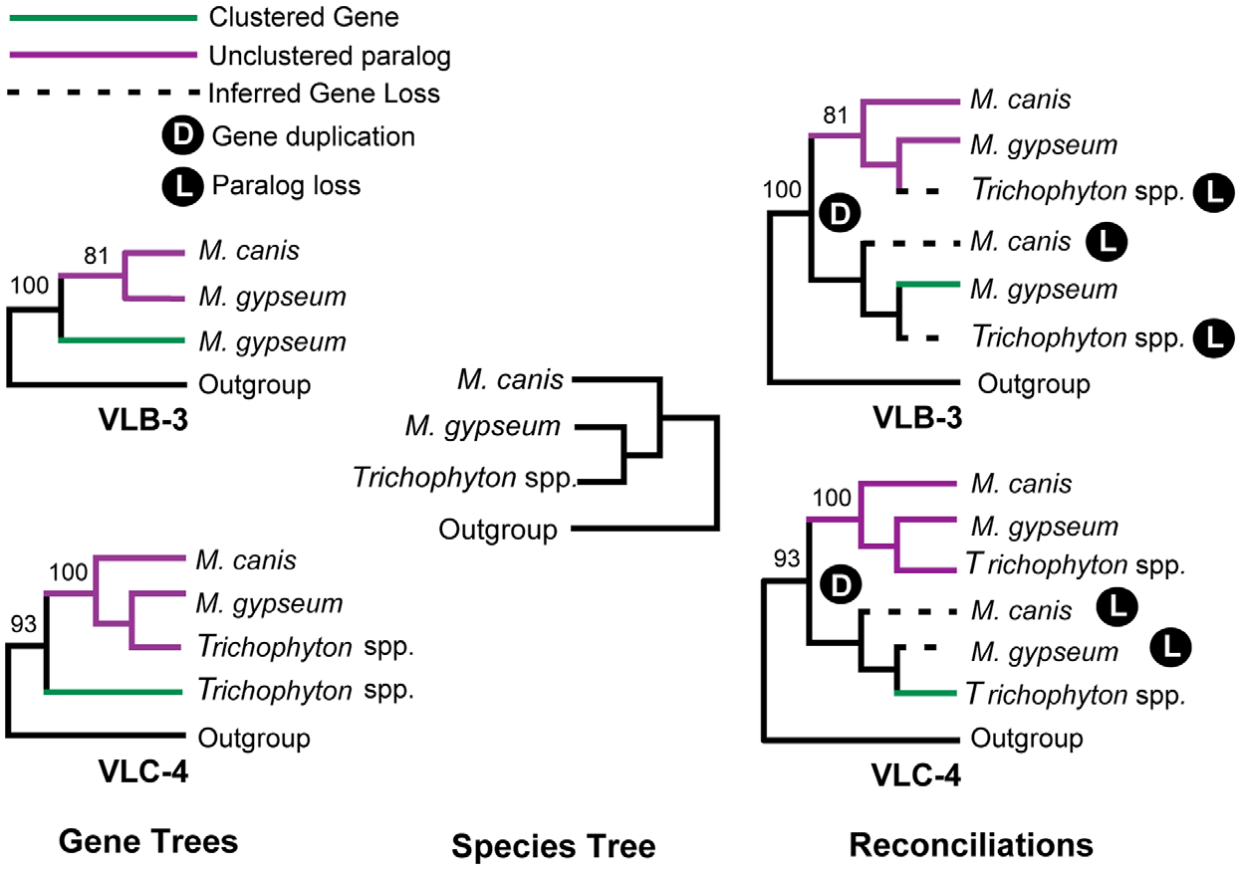
Parsimonious reconstructions of the formation of select VLB and VLC gene families. The left panel shows representative gene trees from VLB (VLB-3) and VLC (VLC-4), which contain recent paralogs. The middle panel displays dermatophyte species relationships. The right panel shows parsimonious reconstructions of duplications and losses required to reconcile each gene tree with the species tree. Maximum likelihood bootstrap support values greater than 60% are shown. Green branches represent sequences clustered in the VL, purple branches represent unclustered paralogs used to infer the ancestral status of genes, and dotted branches represent sequences inferred to have been lost. The VLB-3 reconstruction suggests that both *M. canis* and *Trichophyton* spp. ancestrally contained a VLB-3 ortholog but subsequently lost it, whereas the VLC-4 reconstruction suggests that both *M. canis* and *M. gypseum* ancestrally contained a VLC-4 ortholog but subsequently lost it.

To understand the evolutionary history of the VL conformations, we also reconstructed the evolutionary history of all genes in VLB and VLC. Except for VLB-10 and VLC-12, which are orthologs and shared between VLB and VLC (Figure S2 Z), all other genes are uniquely present in either VLB or VLC. Many of the genes present in the VLB only exist in *M. gypseum*, however, we did find several paralogs residing elsewhere in the *M. gypseum* and the *M. canis* genomes (Figure 3, Figure S3 A). For example, the VLB-2 protein sequence is most closely related with a *M. canis* sequence that is not part of the VL. Similarly, the VLB-3 and VLB-5 sequences are parts of clades containing two pairs of *M. gypseum* and *M. canis* homologs, none of which is part of the VLB or the VL in general. Other genes, including VLB-1, VLB-4, VLB-6, VLB-7, VLB-8, and VLB-9 are found only in *M. gypseum* and have little or no support for phylogenetic placement. All clustered porphyrin metabolism genes have unclustered homologs in other fungal genomes (Figure S2).

The distribution of the VLC genes is somewhat complementary to those in VLB in that many of the genes present in the VLC only exist in *Trichophyton* spp. For example, the VLC-2, VLC-3, VLC-6, and VLC-7 genes are only present in *Trichophyton* spp. and are absent from *M. canis* and *M. gypseum*. VLC-4 and VLC-5 sequences each group with clades comprised of sequences that are distributed uniformly among dermatophytes (VLC-4) or missing only from *M. canis* (VLC-5), and whose inferred phylogeny is concordant with expected species relationships [27]. Gene – species tree reconciliation suggests that the orthologs of VLC-4 and VLC-5 were ancestrally present in all seven dermatophytes but were subsequently lost from *M. canis* and *M. gypseum* (Figure S3 C). VLC-1 and VLC-8 belong to the same gene family and although they are only present in *Trichophyton* spp., they both group with clades comprised of sequences distributed uniformly among dermatophytes and that are largely consistent with species relationships. Similar to VLC-4 and VLC-5, gene – species tree reconciliation suggests that VLC-1 and VLC-8 were present in the dermatophyte ancestor. The VLC-9 sequence also groups with clades that are uniformly distributed in dermatophyte genomes and consistent with species relationships, and one whose orthologs in *M. canis* and *M. gypseum* appear to have been lost (Figure S3 D).

### Homologs of VL Genes are Clustered in Other Loci and Lineages

Even though only one gene is shared between VLB and VLC, the *M. gypseum* genome also contains a gene cluster comprised of VLC-1/VLC-8 and VLC-9 homologs that resides outside the VL. Specifically, a *M. gypseum* sequence (T1) closely related to VLC-1/VLC-8 and a *M. gypseum* sequence (T2) closely related to VLC-9 form a two gene cluster elsewhere in the *M. gypseum* genome (Figure S3 D). Similarly, homologs of VLB-10 and VLB-11 are found clustered in *Penicillium chrysogenum*. Interestingly, in the VLB-10 and VLB-11 phylogenies the *M. gypseum* and *P. chrysogenum* were supported to be sister sequences to the exclusion of other dermatophyte sequences (Figures S2 O and P), raising the hypothesis that these two genes were likely acquired via horizontal gene transfer. Clustering of porphyrin metabolism genes is unique to *M. gypseum* in the fungi.

## Discussion

**Figure 4:**
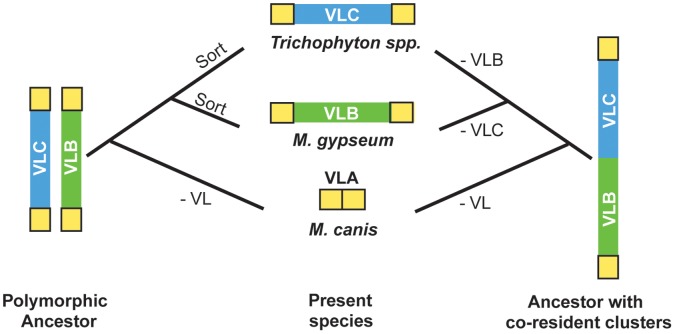
The “polymorphism” and “co-residence” scenarios are two alternative hypotheses for the evolution of the VL. The loci represented on the left and right of the figure depict alternative ancestral states. Species phylogenies are labeled with the minimal events required to generate the VLA, VLB, and VLC conformations described in this study.

We identified three distinct conformations in a variable locus among seven dermatophyte genomes from species of the family Arthrodermataceae. Whereas the first conformation does not contain any protein-coding sequences, the other two contain gene sets that are characteristic of NRPS secondary metabolism gene clusters [14], [28]. Furthermore, evolutionary analyses show that genes residing in the two clusters have very different evolutionary histories than most of their neighbors located in the flanking regions. Specifically, whereas the genes flanking the VL are largely vertically inherited single-copy orthologs, the VL genes appear to be parts of well-diversified gene families, whose evolution has been shaped by several different processes, including gene duplication and horizontal gene transfer (Figure 4, Figure S2 A–AJ) [6]–[10], [29]–[32].

### The VLB Gene Cluster is Likely Involved in Porphyrin Metabolism and Iron Acquisition

Skin is a notoriously difficult substrate from which to acquire iron [3], and both porphyrins and fungal siderophores made by NRPS genes are used in iron homeostasis [13], [33]. The functional annotation of the four porphyrin genes in VLB (VLB-3: porphobilinogen deaminase; VLB-5: porphobilinogen synthase; and VLB-6/9∶5-aminolevulinate synthases) suggests that the gene cluster is involved in the production of porphyrin compounds from glycine in accordance with KEGG Pathway 00860. Intriguingly, glycine is the largest amino acid component of human epidermal keratin [34], so the gene cluster’s predicted function may be relevant to the opportunistic colonization of skin. Furthermore, *M. gypseum* infections fluoresce differently from *M. canis* and most of the *Trichophyton* spp. dermatophytes under wood’s lamp [35], which is a useful diagnostic criterion, and we speculate that a porphyrin ligand produced by the VLB cluster could be responsible for this difference in phenotype.

Clustering of porphyrin metabolism genes is widespread in bacteria [36], but to our knowledge this is the first report of a putative porphyrin metabolism gene cluster in fungi. Interestingly, gene clusters involved in the production of siderophores in some bacteria contain porphyrin metabolism genes along with NRPS genes [37], [38], such as is seen in the VLB. The co-occurrence of the porphyrin metabolism genes with genes typical of secondary metabolism gene clusters raises the possibility that part of this secondary metabolism gene cluster was evolutionarily assembled from primary metabolic pathway components.

### VLB and VLC Gene Clusters are Ancestral but were Lost in Alternative Lineages

Reconstructions of gene duplication and loss events provide evidence that orthologs of many VLC and VLB cluster genes were present in the common ancestor of the Arthrodermataceae dermatophytes, arguing that the entire VLB and VLC gene clusters were present in the dermatophyte ancestor but were subsequently differentially lost or retained in different lineages. Two alternative scenarios can explain this pattern, namely co-residence and polymorphism (Figure 4).

Under the “co-residence” scenario, the VL in the genome of the dermatophyte ancestor contained both the VLB and the VLC clusters on the same chromosomal haplotype, and different deletions in each of the three main lineages resulted in the present distribution. Specifically, the entire VL locus was deleted in *M. canis*, whereas all but the last gene of the VLB was deleted in *Trichophyton* spp. and all but the last gene of the VLC was deleted in *M. gypseum*.

Under the “polymorphism” scenario, the VL in the genomes of members of a sexually recombining population of the dermatophyte ancestor was polymorphic for the two alternative states VLB and VLC. During subsequent evolution, the entire VL was deleted in *M. canis*, whereas the VLB and VLC “alleles” were differentially sorted in *M. gypseum* and *Trichophyton* spp., respectively. Multigene polymorphism has been demonstrated previously in fungi [39], [40], as well as in animals [41]. For example, the *GAL* gene cluster, which enables the utilization of galactose, is present in two different versions in *Saccharomyces kudriavzevii* [40]. However, whereas the *GAL* locus contains functional and non-functional alleles of the same genes, the alleles in the case of the VL polymorphism, if true, would have contained non-homologous multigene segments. Discriminating which of the two alternative scenarios is more likely to explain the origin of the variable locus will require additional genome sequencing within and between species of Arthrodermataceae.

## Supporting Information

Figure S1 **Genome alignment of the ∼100 Kb region that includes the VL.** Nucleotide sequences corresponding to the genomic region encompassing the VL locus in available dermatophyte genomes were aligned using the Mauve software. The dotted line demarks the VL, and the yellow, green, and blue bars on the left denote genomes with the VLA, VLB, and VLC conformations, respectively. The *Arthroderma benhamiae* sequence was derived from two supercontigs: Supercontig 28 contains the left flank, the VL, and approximately 10 kb of the right flank, and supercontig 35 contains the remainder of the sequence that corresponds to the right flank in the other species.
(PDF)

Figure S2 **Phylogenetic trees of proteins encoded by the VL and flanking regions.** (A–F) Highlighted sequence names labeled with “L1–6” are orthologs of genes on the 5′ flank of the VL, and (AA–AJ) highlighted sequence names labeled with “R1–12” are orthologs of genes on the 3′ flank of the VL. R5 and R6 are genes predicted in *M. gypseum* that were excluded from the analysis due to absence of orthologous sequences from other genomes. (G–Z) VLB and VLC gene orthologs are highlighted in green and blue respectively. Maximum Likelihood support values for nodes greater than or equal to 60% are shown.
(PDF)

Figure S3 **Parsimonious reconstructions of all VLB and VLC gene families.** (A) Reconstructions of gene duplication and loss in VLB gene families. (B) Reconstructions of gene duplication and loss in VLC gene families. (C) Reconstructions of gene duplication and loss in multigene families containing more than one paralog in the VL. Genes VLC-8, VLC-1, and T1 are in the same gene family and are displayed on the left panel. Genes T2 and VLC-9 are in the same gene family and are on the right panel. VLC-8 and VLC-9 are physically clustered in *Trichophyton* spp. genomes, whereas their paralogs T1 and T2 are physically clustered in the *M. gypseum* genome in another locus outside the VL. Maximum likelihood bootstrap support values greater than 60% are shown. Green branches represent sequences clustered in the VL, purple branches represent unclustered paralog sequences used to infer the ancestral status of genes, dotted branches represent sequences inferred to have been lost, and red branches represent sequences inferred to have been acquired via horizontal gene transfer.
(PDF)

Table S1 **Complete and draft genomes analyzed in this study.**
(PDF)
