# Supplementary figures and images for: Two Different Secondary Metabolism Gene Clusters Occupied the Same Ancestral Locus in Fungal Dermatophytes of the Arthrodermataceae
Source: PLoS One. 2012 Jul 30;7(7):e41903. doi: 10.1371/journal.pone.0041903 (PMC3408471; doi:10.1371/journal.pone.0041903)

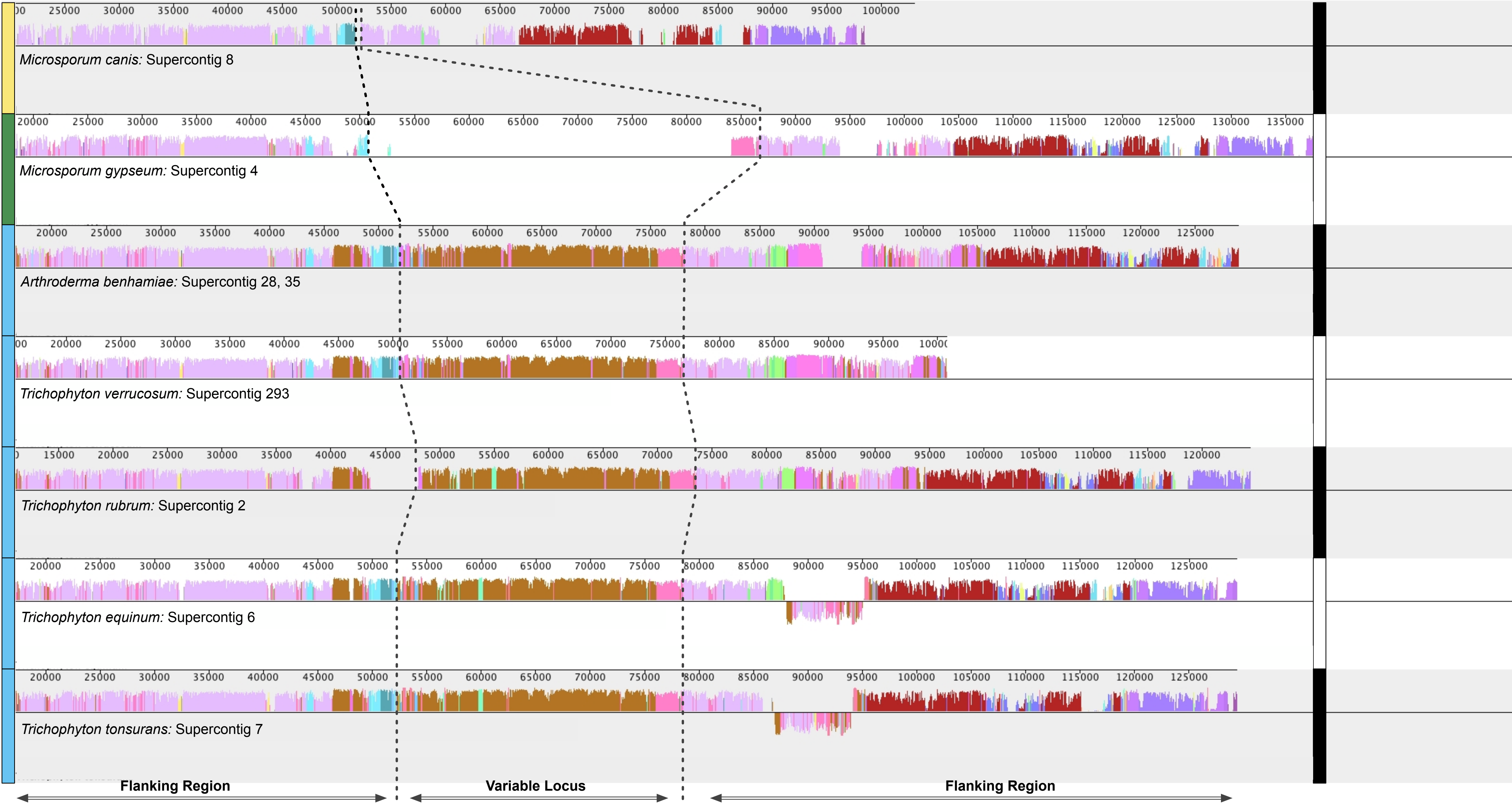

Supplement: Figure S1 — Genome alignment of the ∼100 Kb region that includes the VL. Nucleotide sequences corresponding to the genomic region encompassing the VL locus in available dermatophyte genomes were aligned using the Mauve software. The dotted line demarks the VL, and the yellow, green, and blue bars on the left denote genomes with the VLA, VLB, and VLC conformations, respectively. The Arthroderma benhamiae sequence was derived from two supercontigs: Supercontig 28 contains the left flank, the VL, and approximately 10 kb of the right flank, and supercontig 35 contains the remainder of the sequence that corresponds to the right flank in the other species. (PDF) [file pone.0041903.s001.pdf]

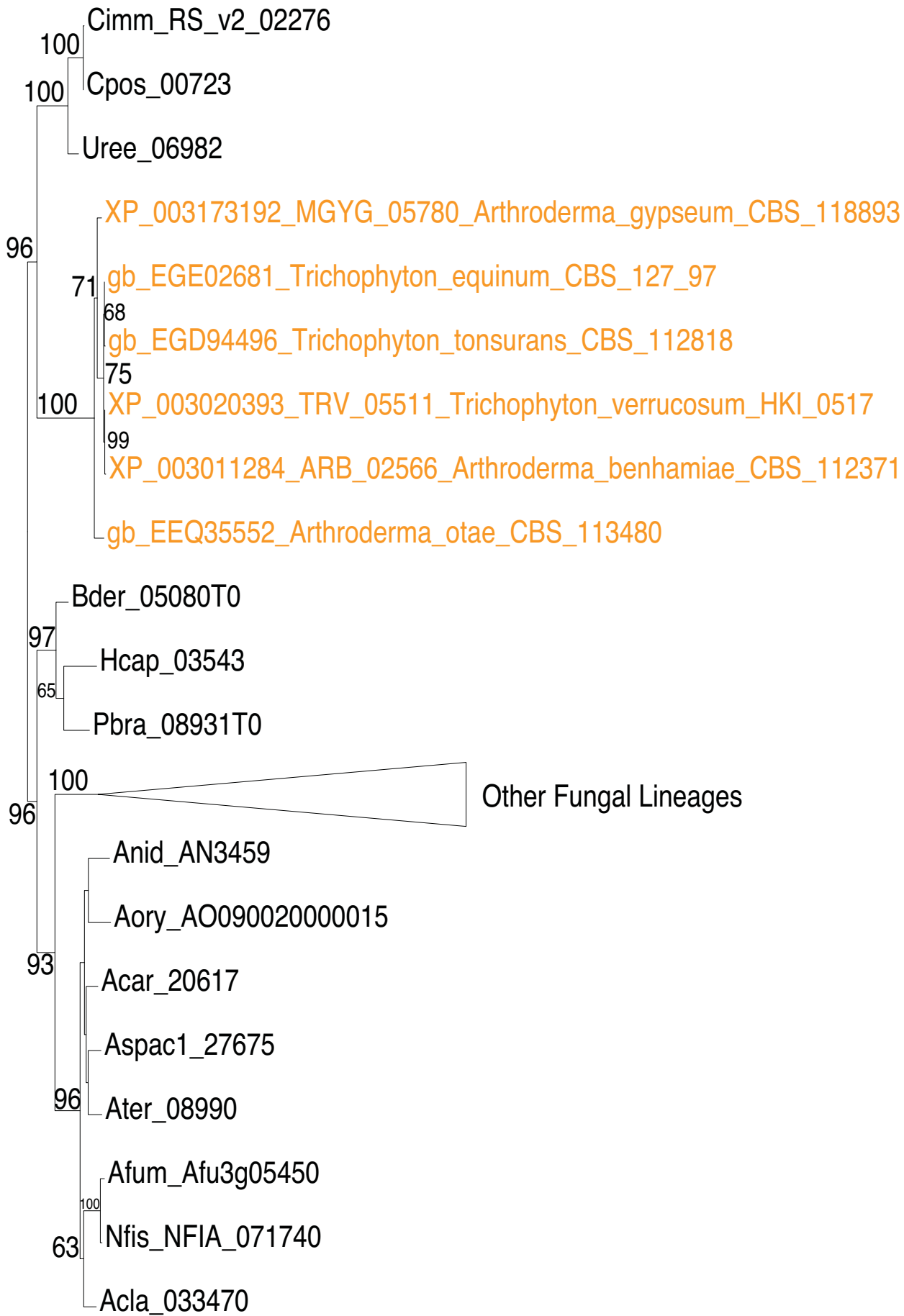

0.4

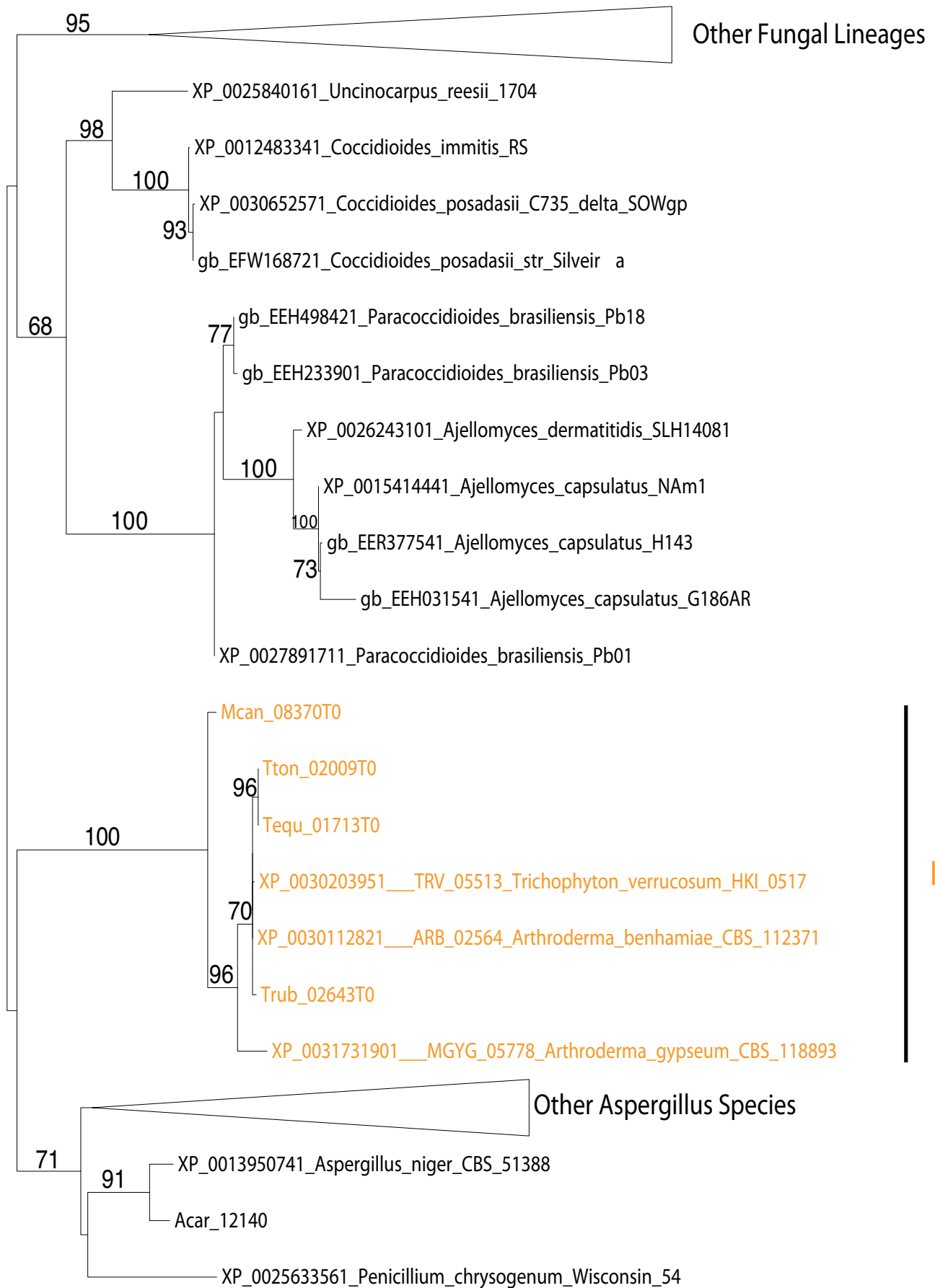

L2

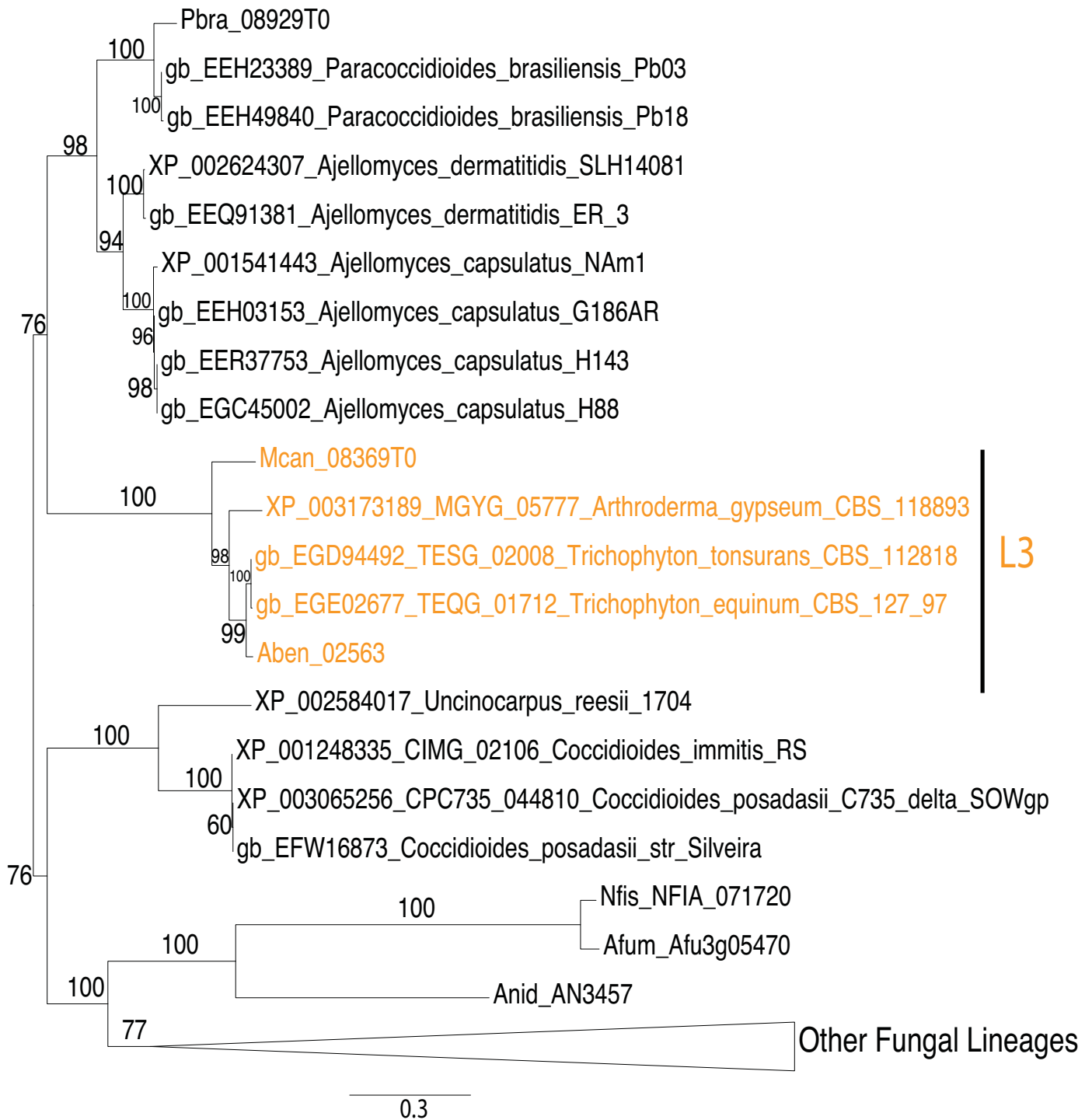

## Other Fungal Lineages

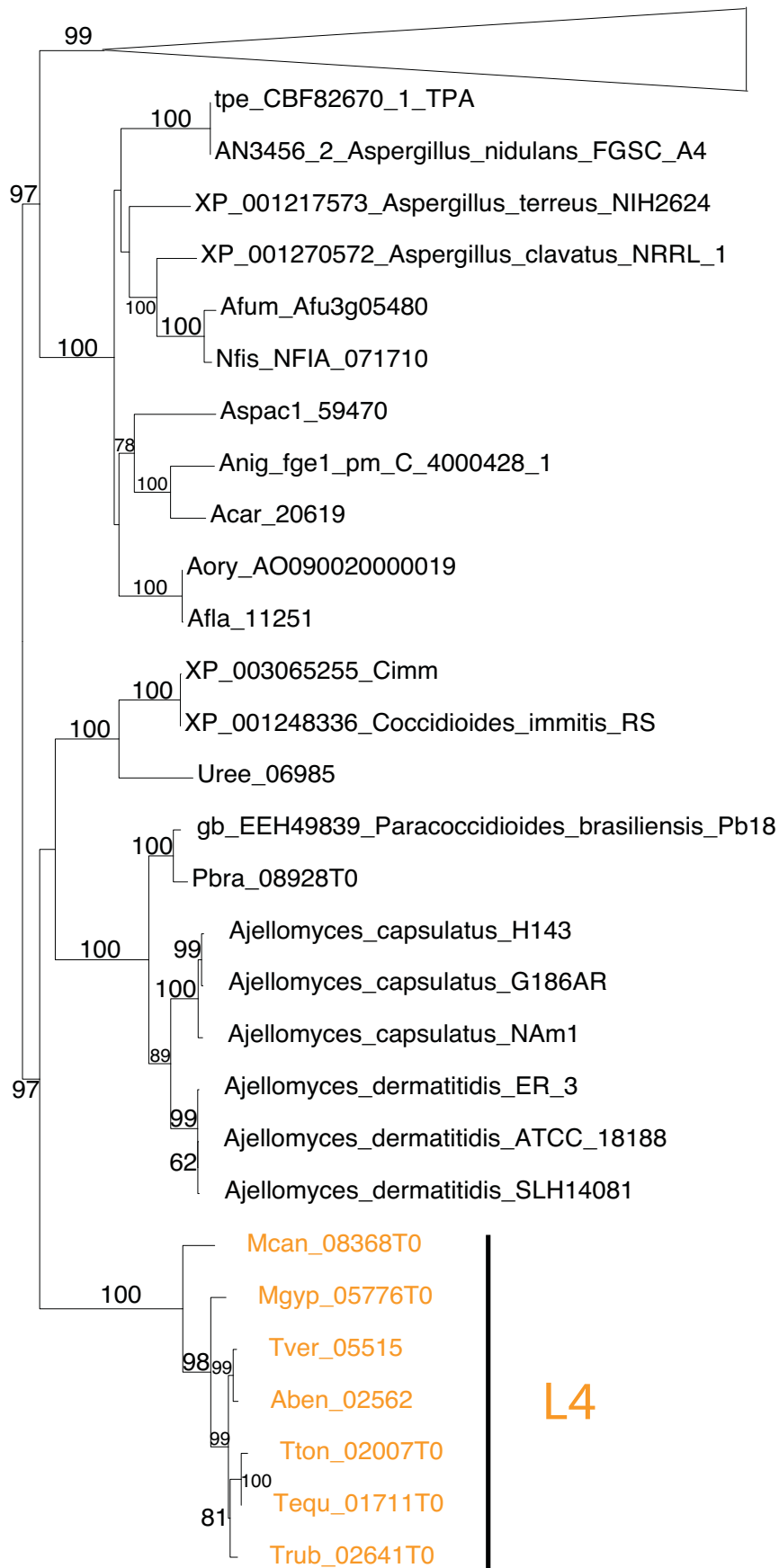

## L4

0.4

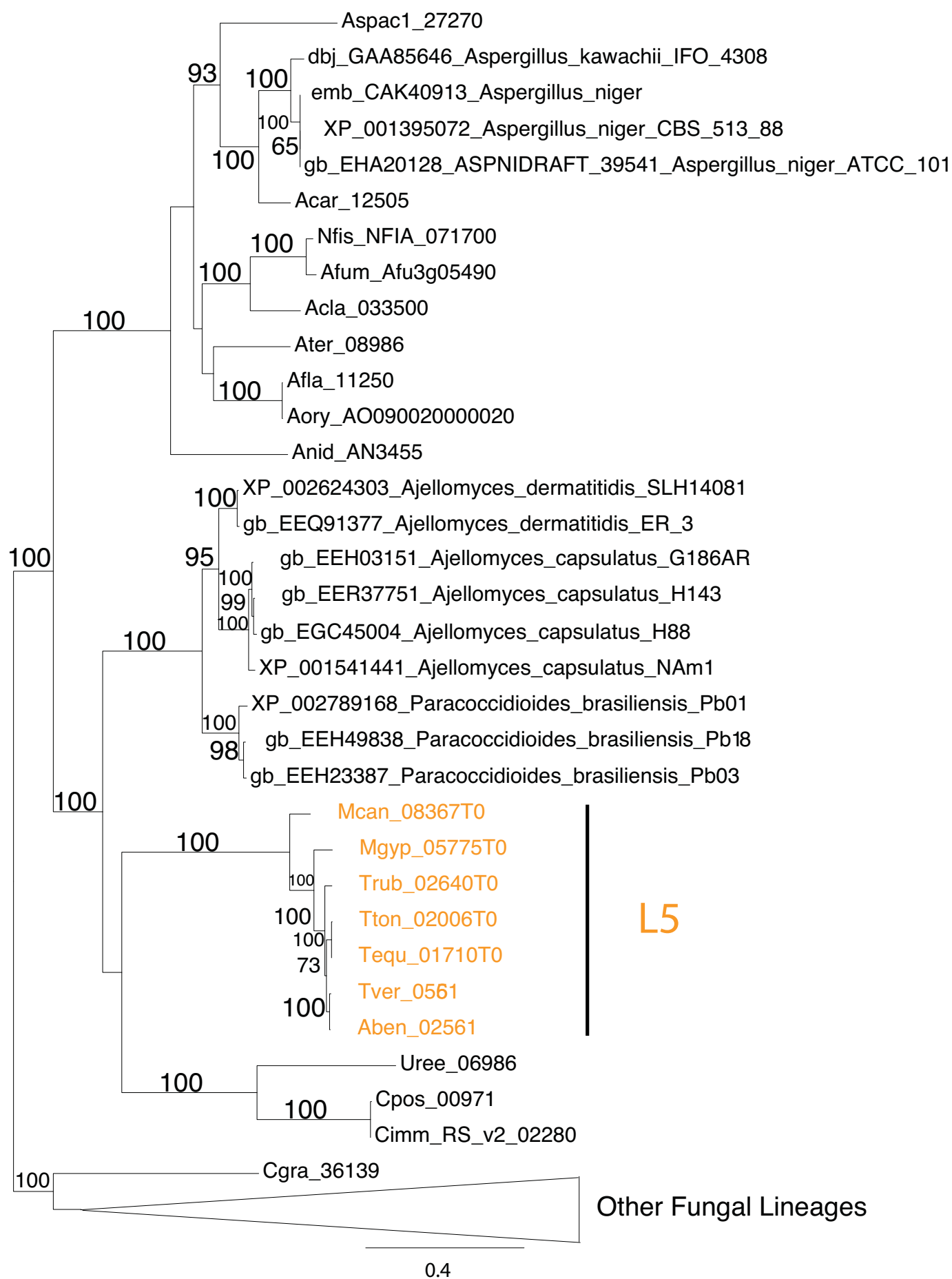

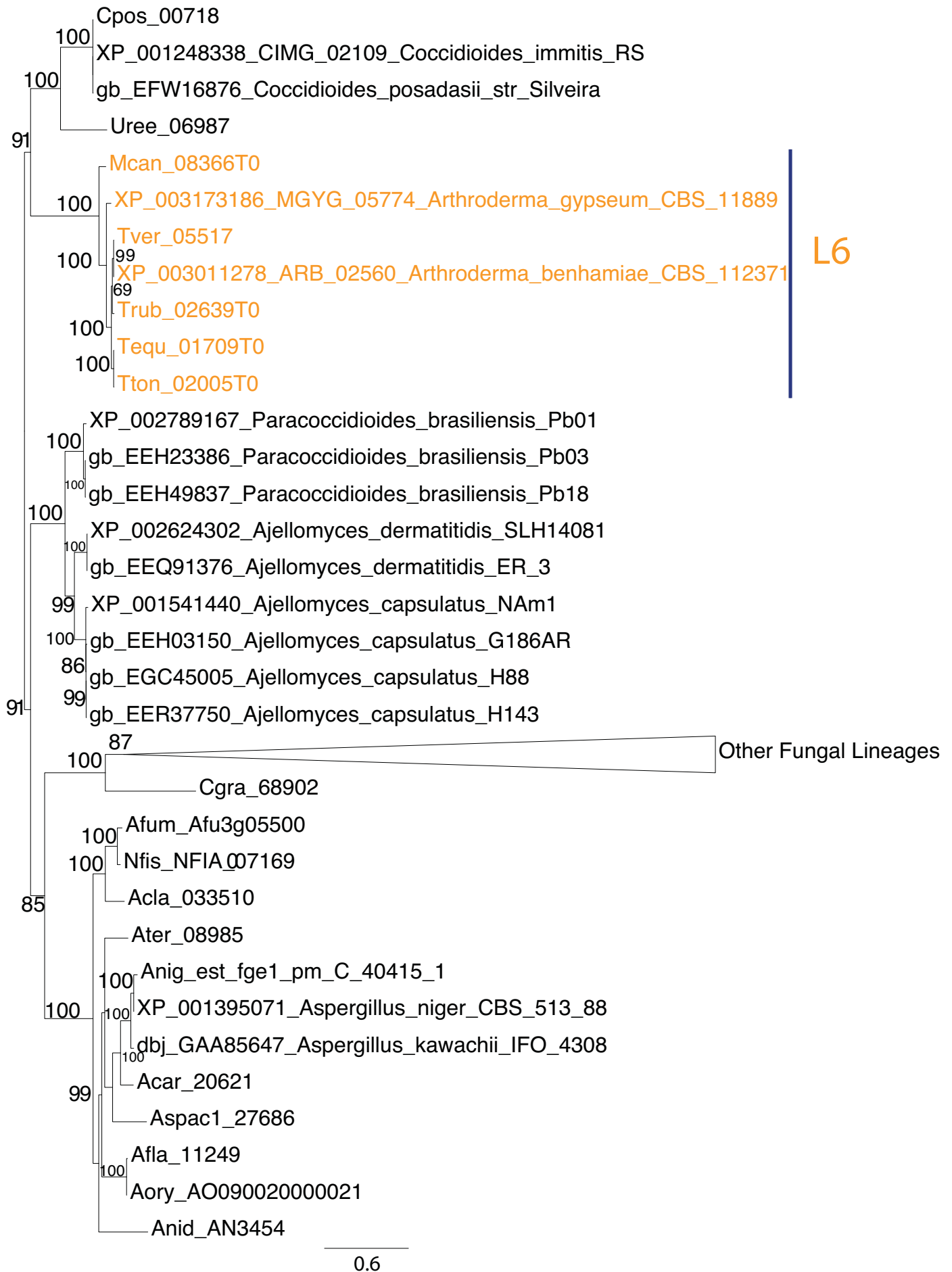

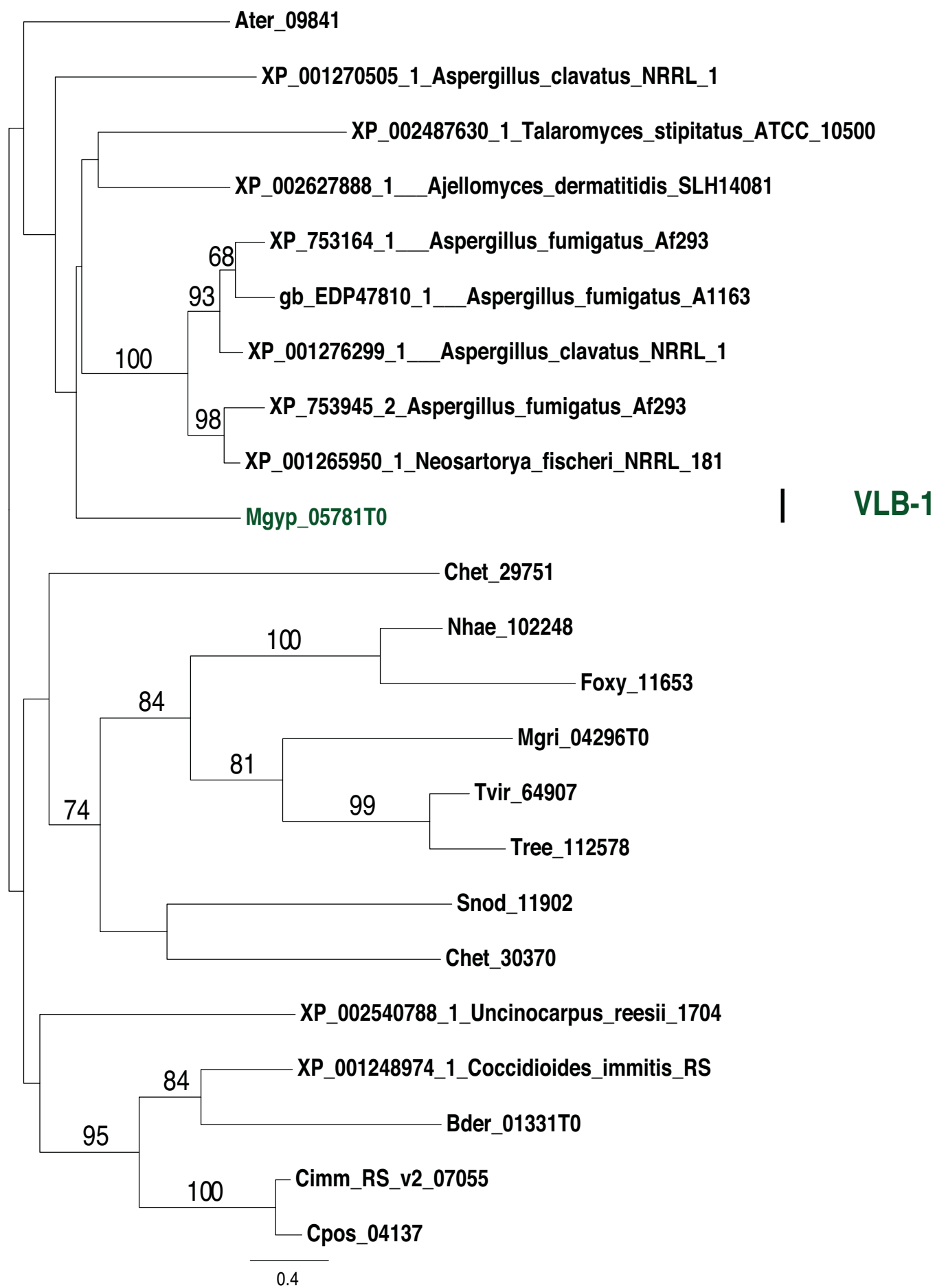

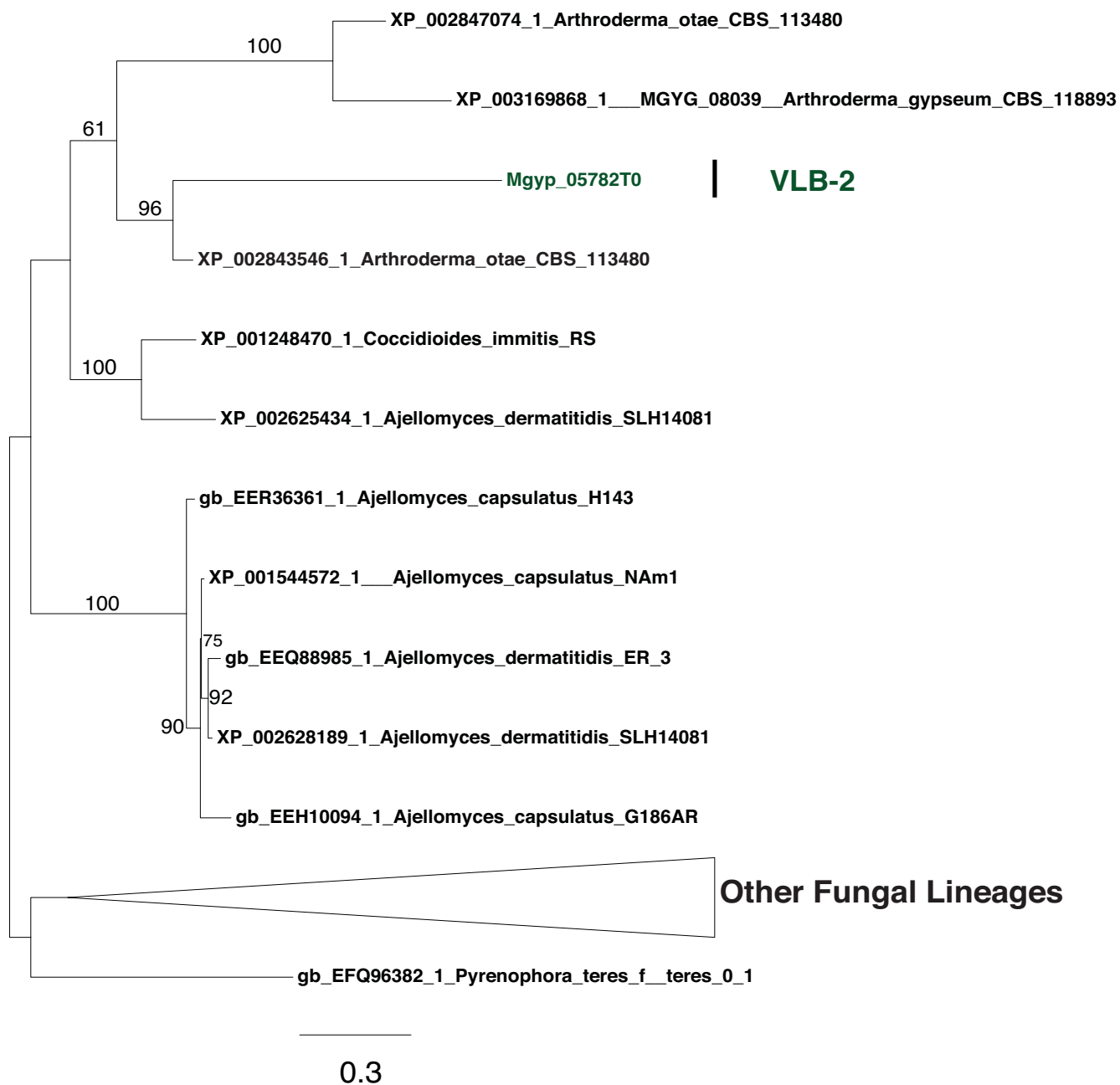

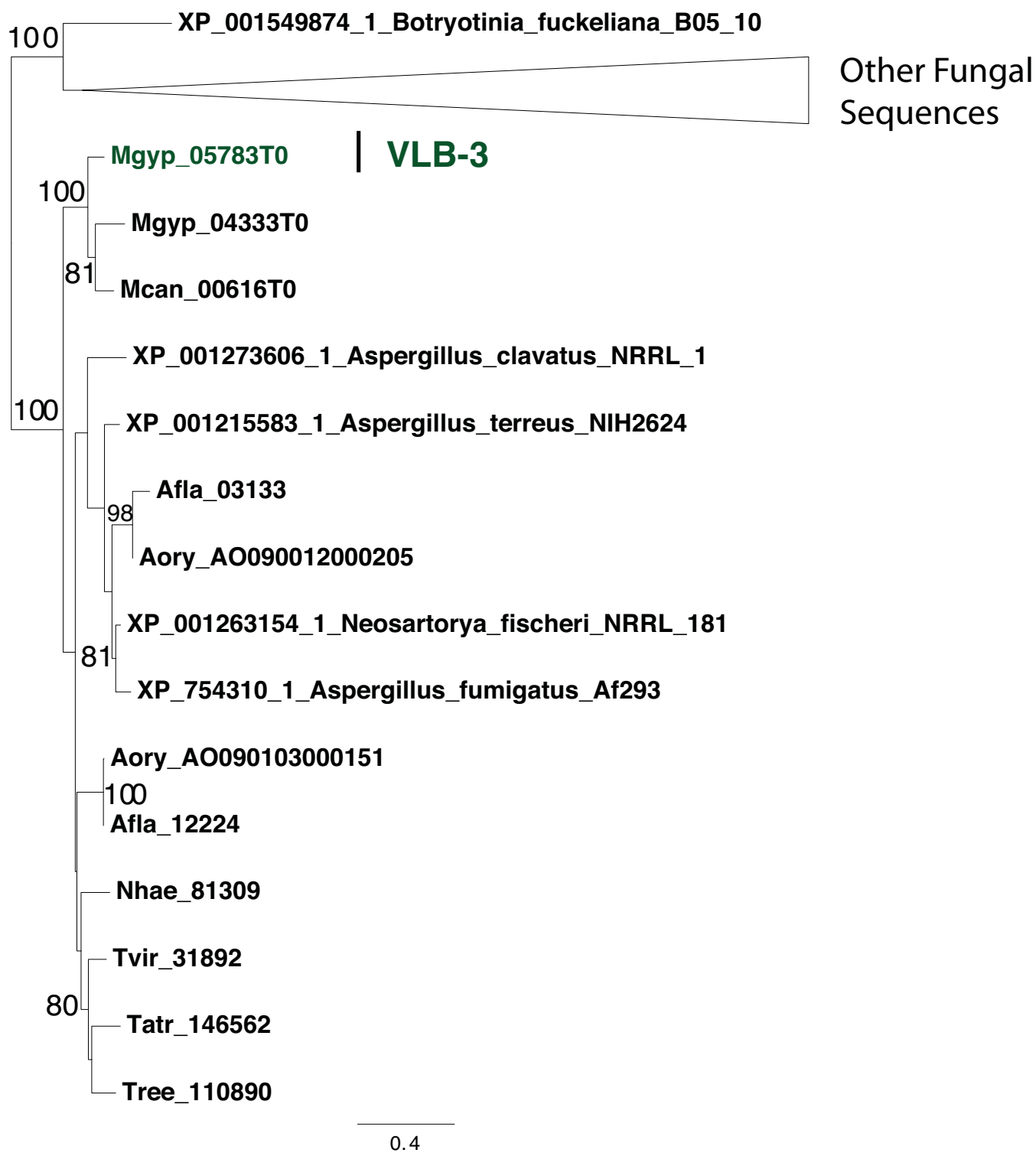

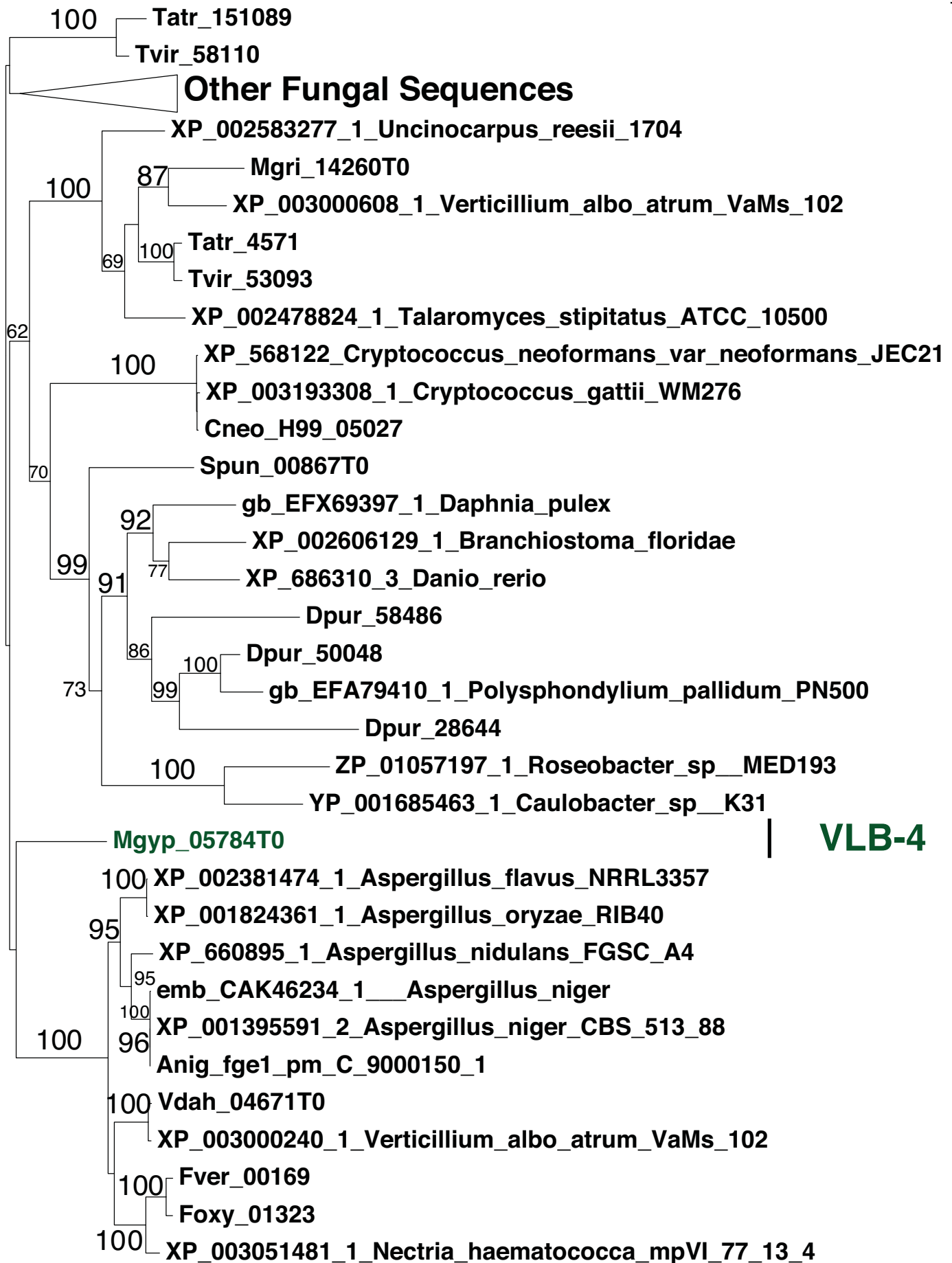

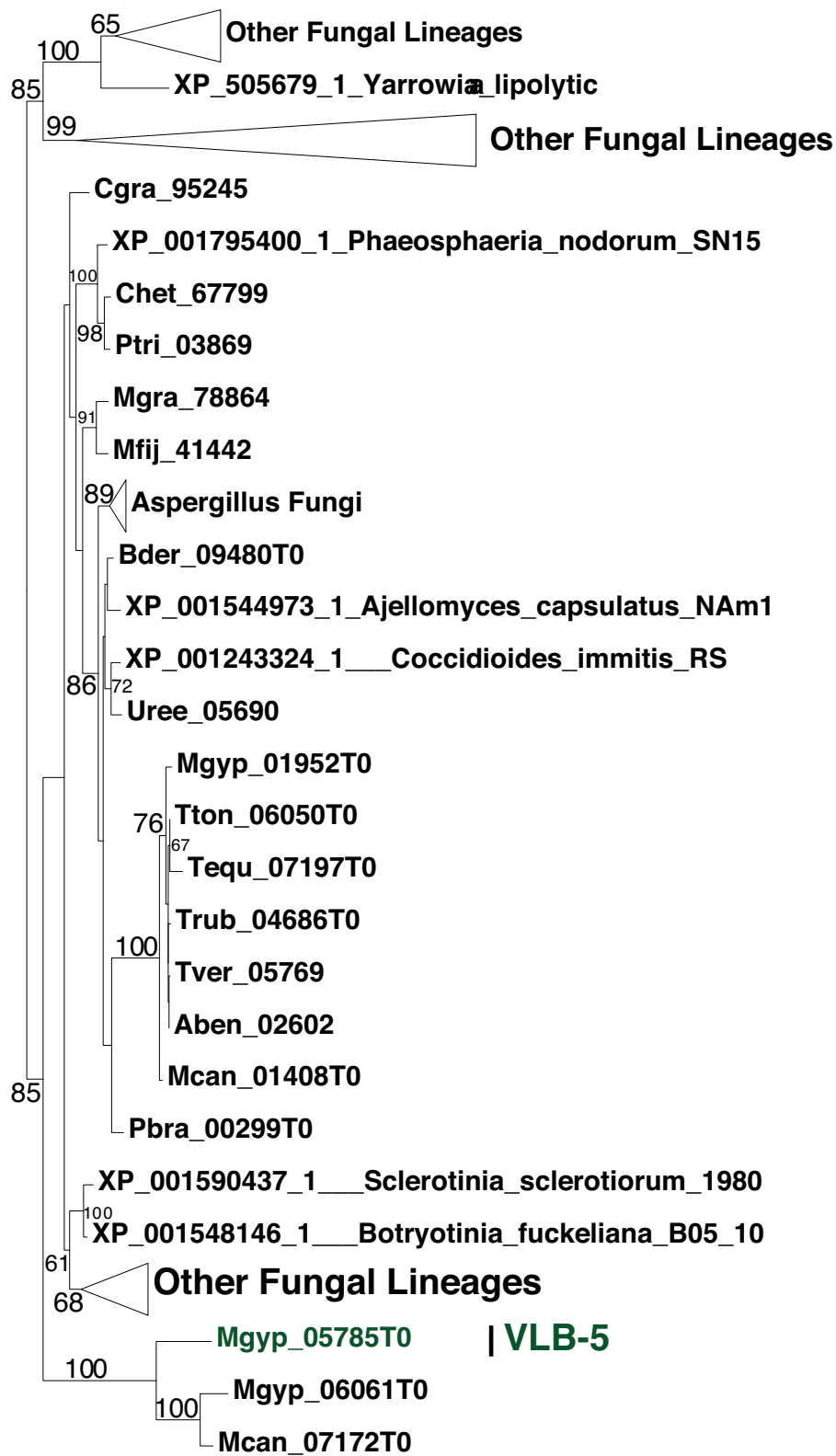

| VLB-5

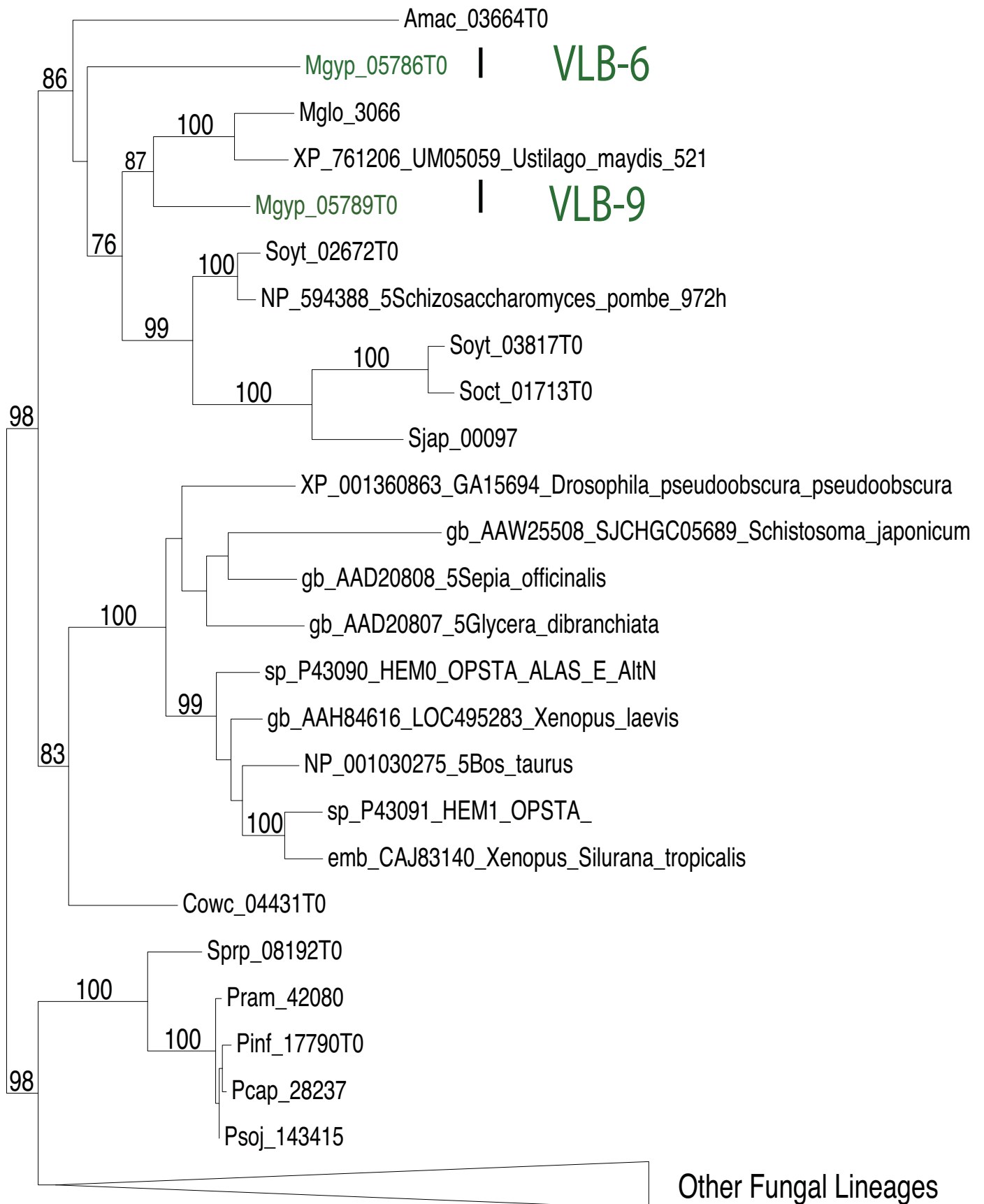

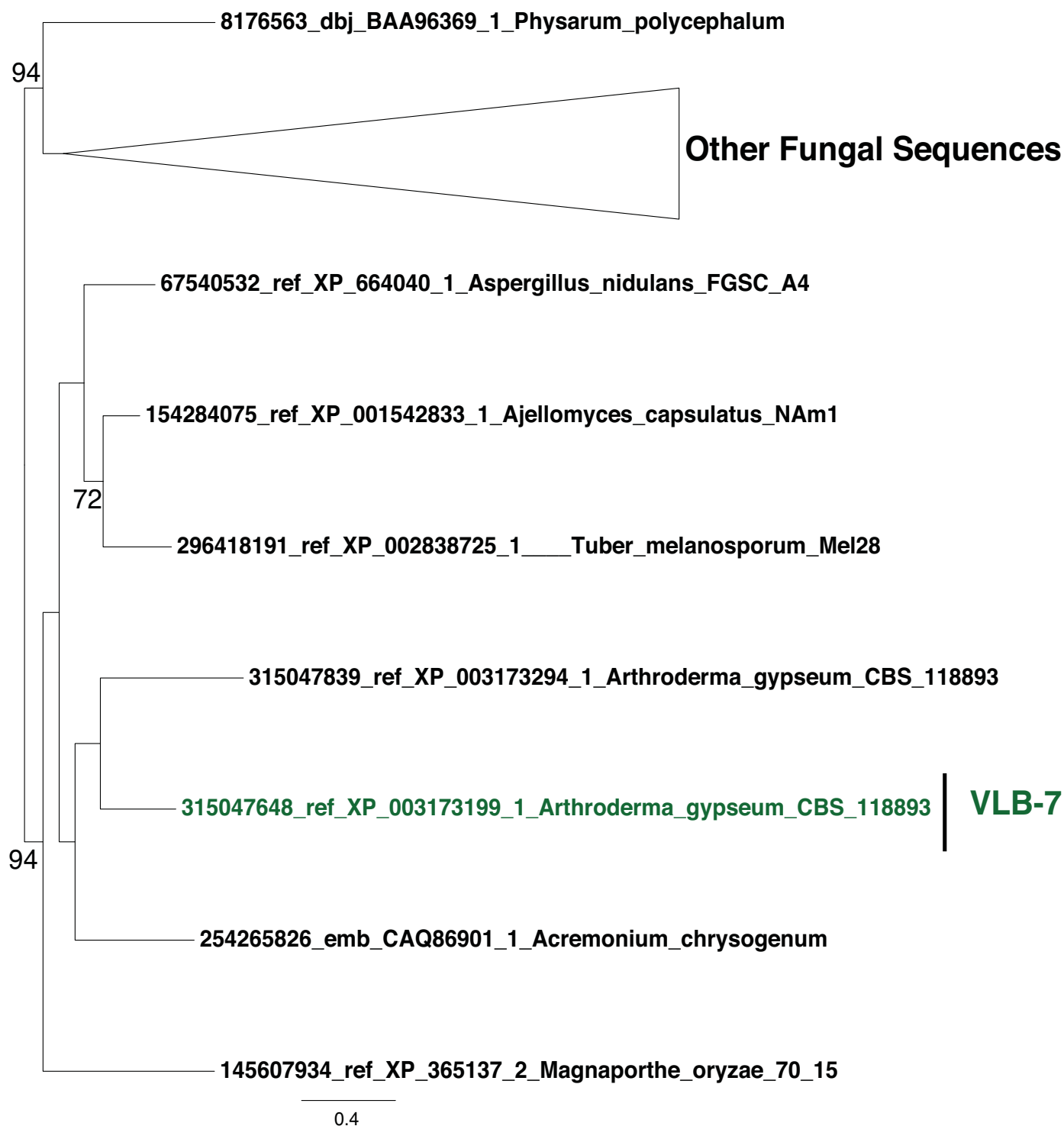

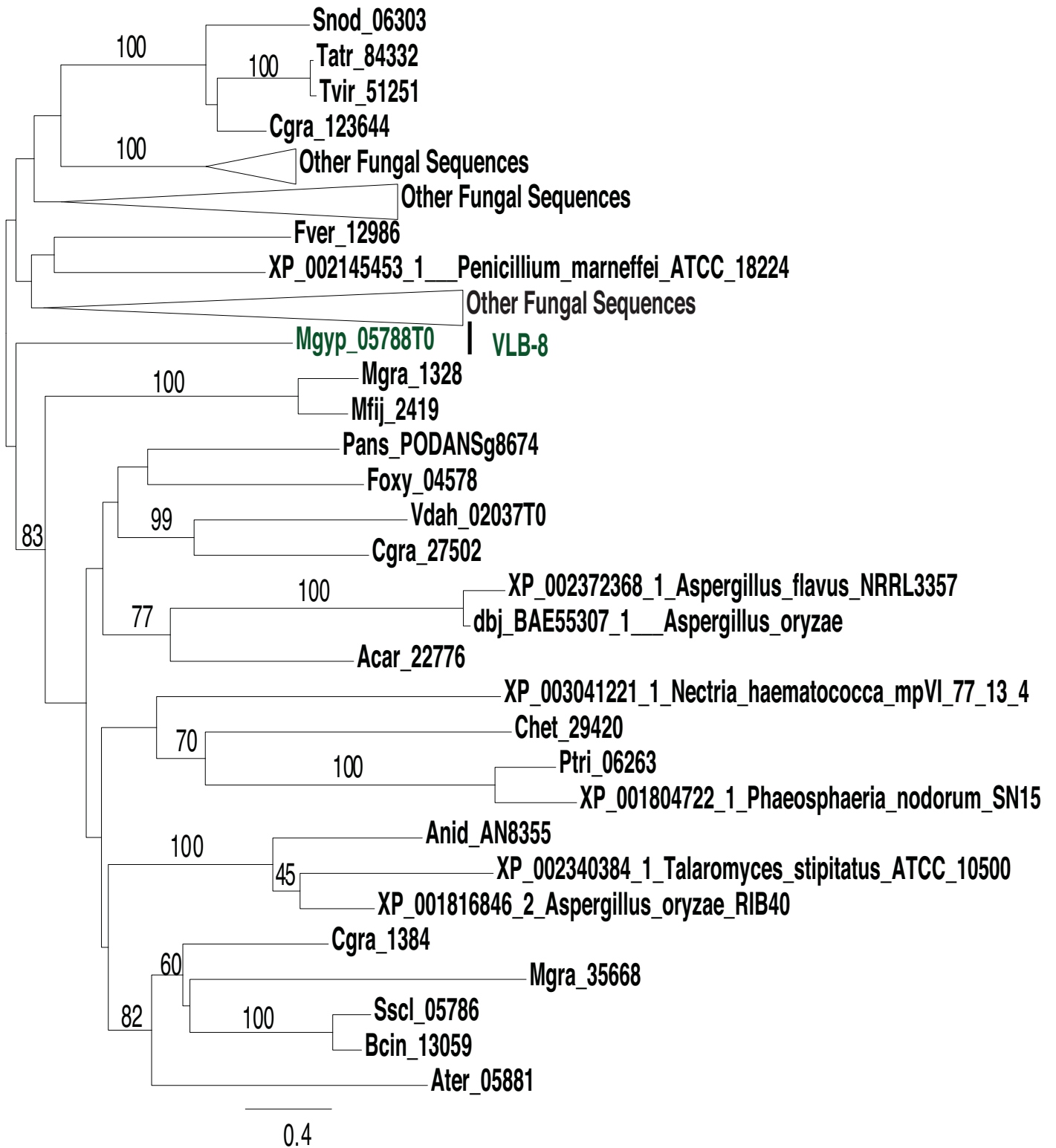

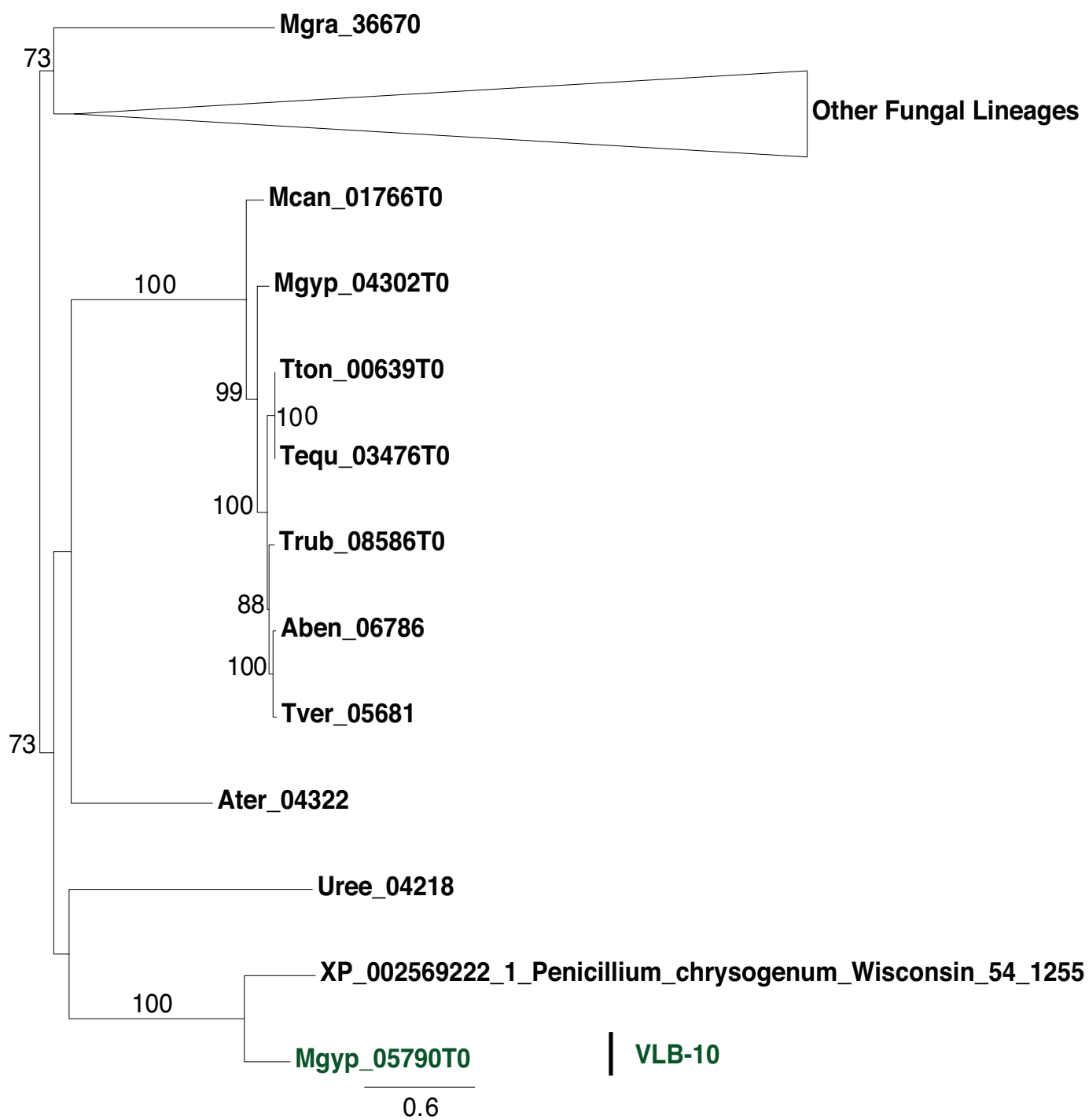



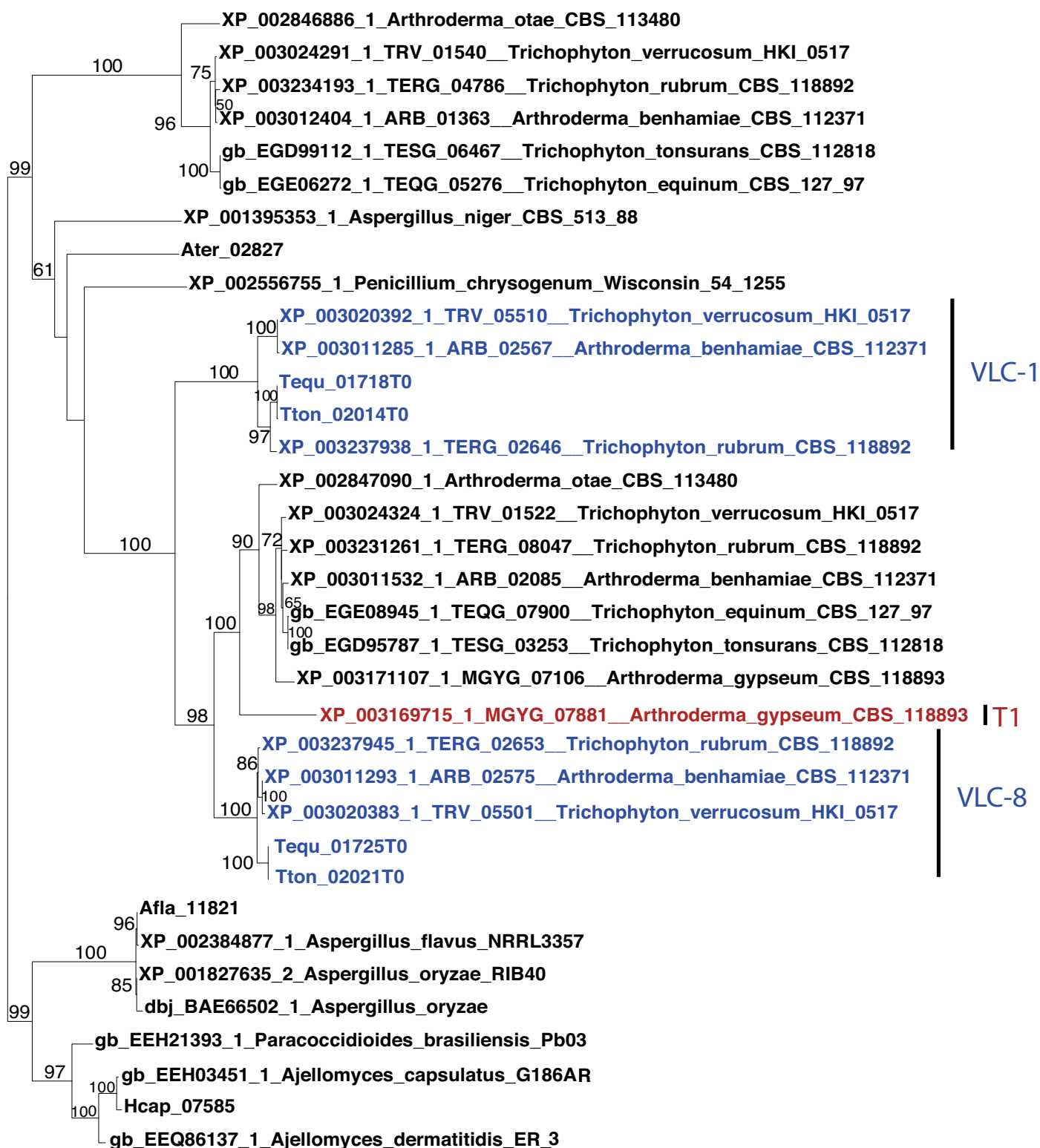

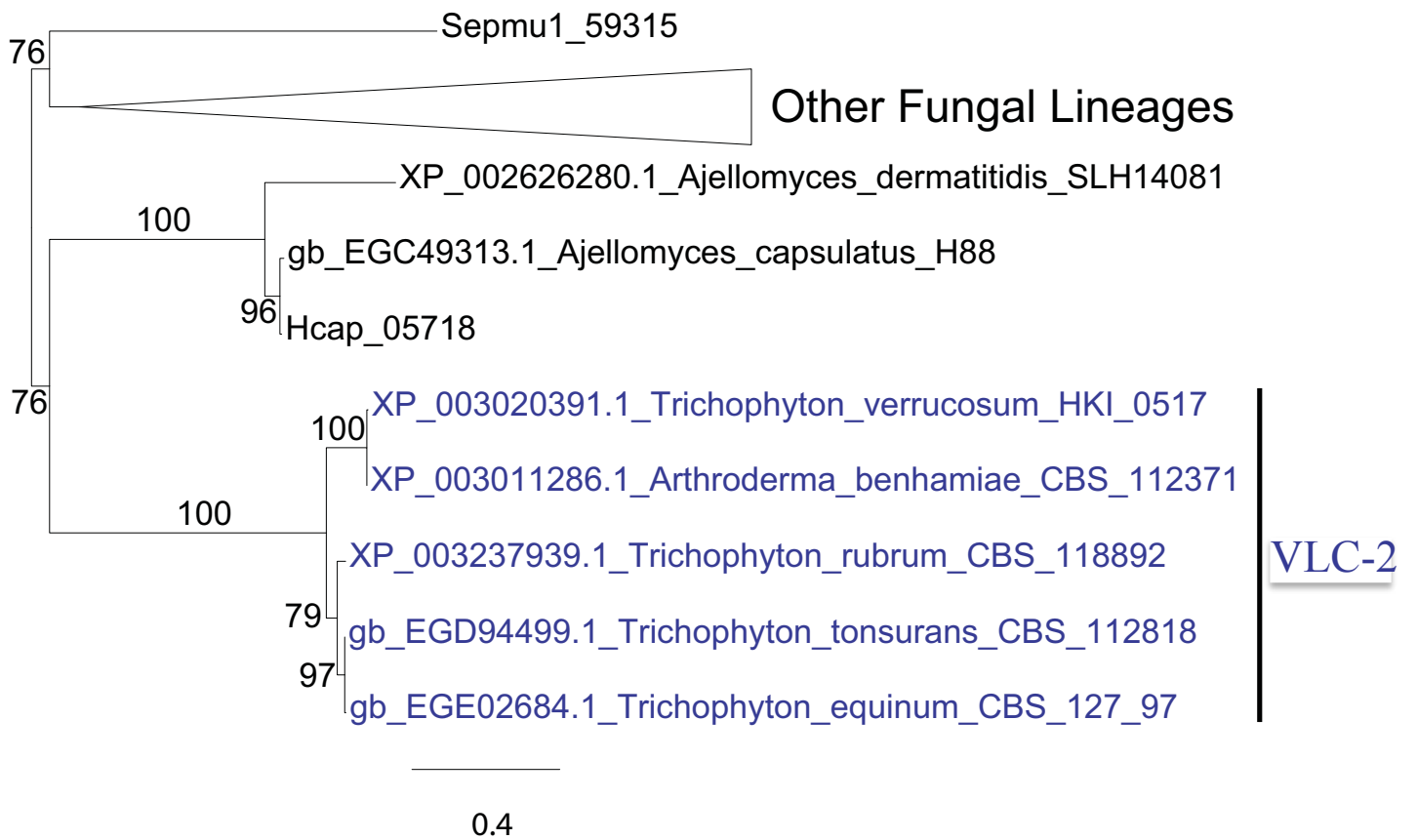

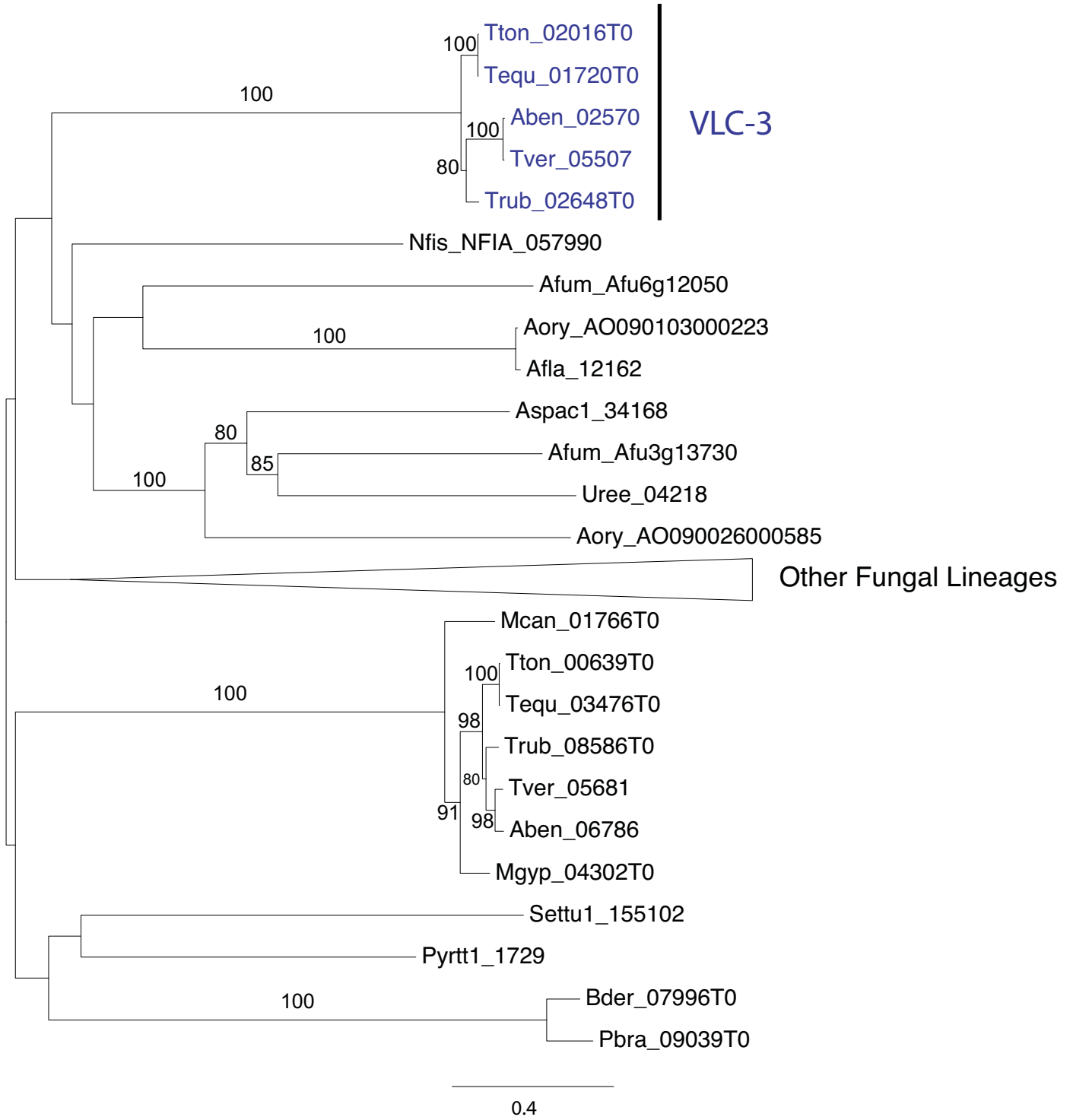

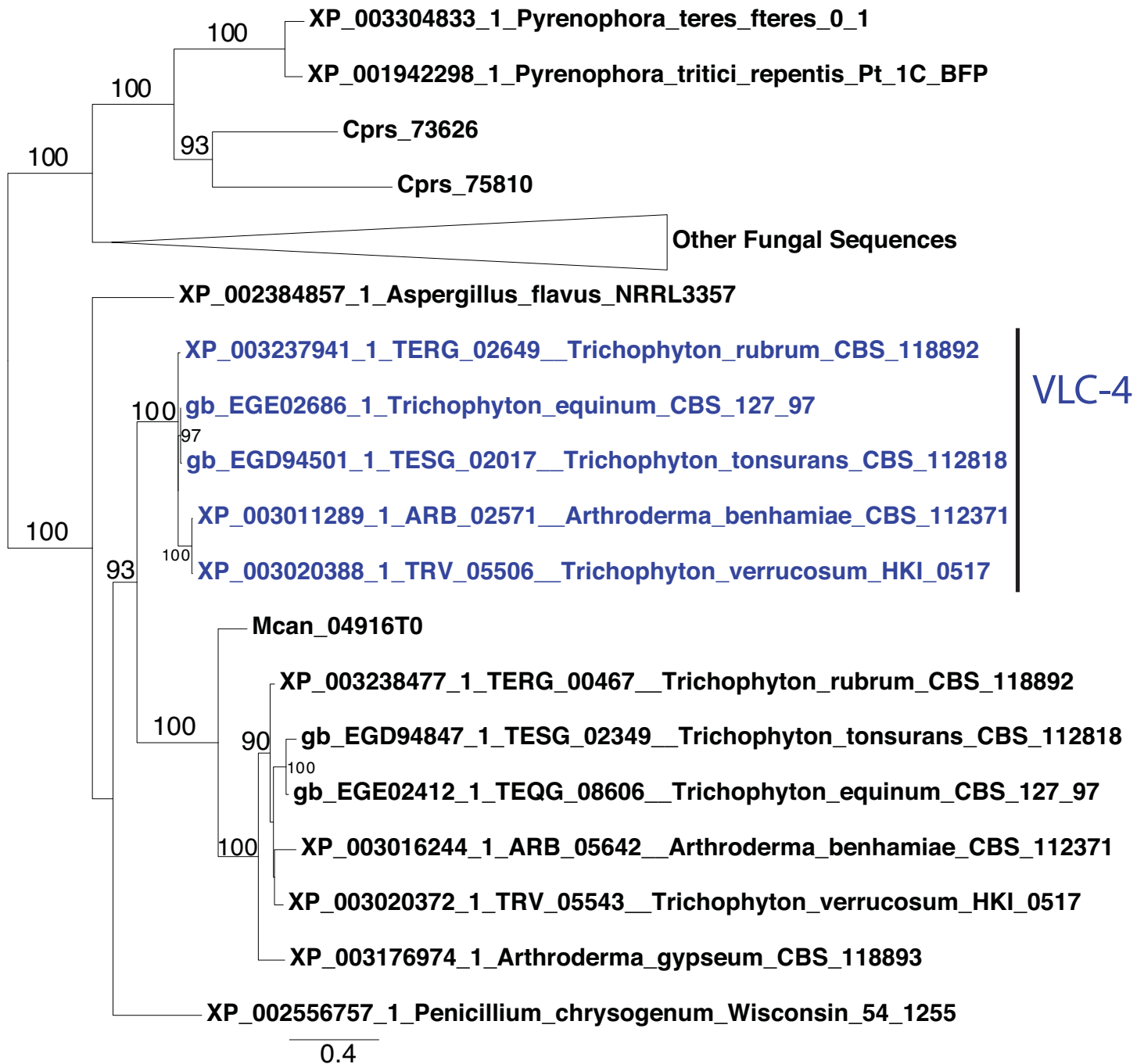

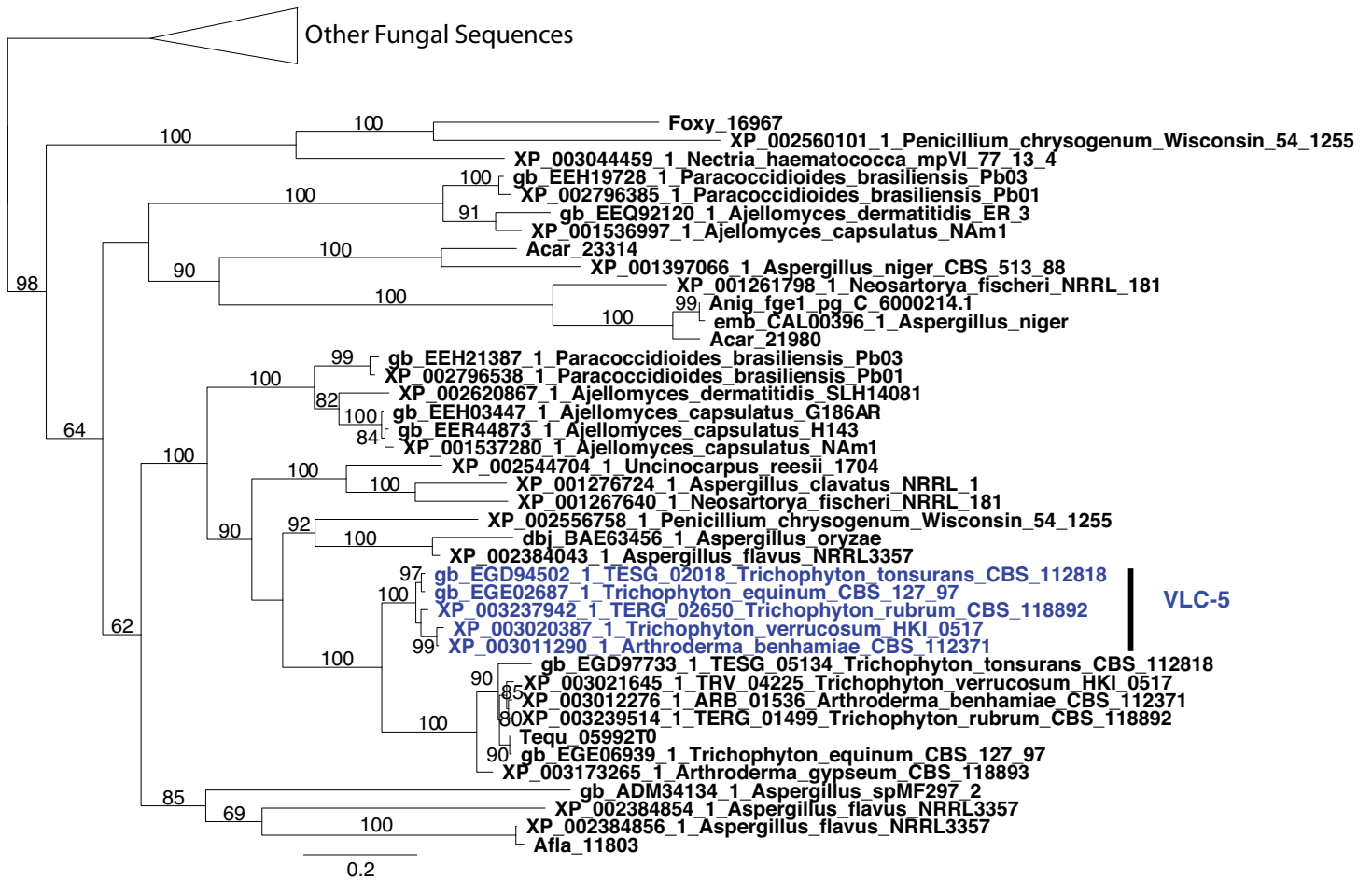

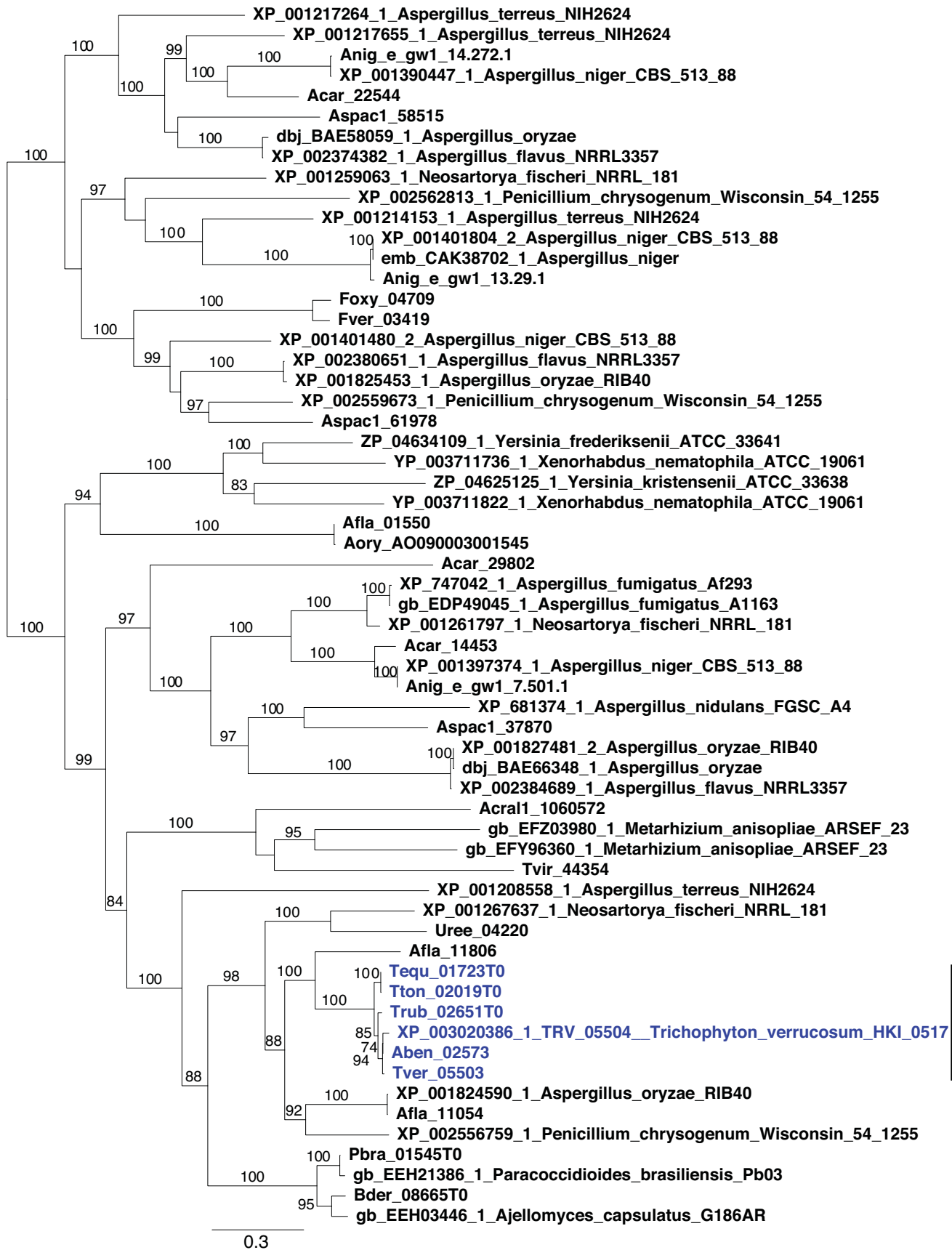

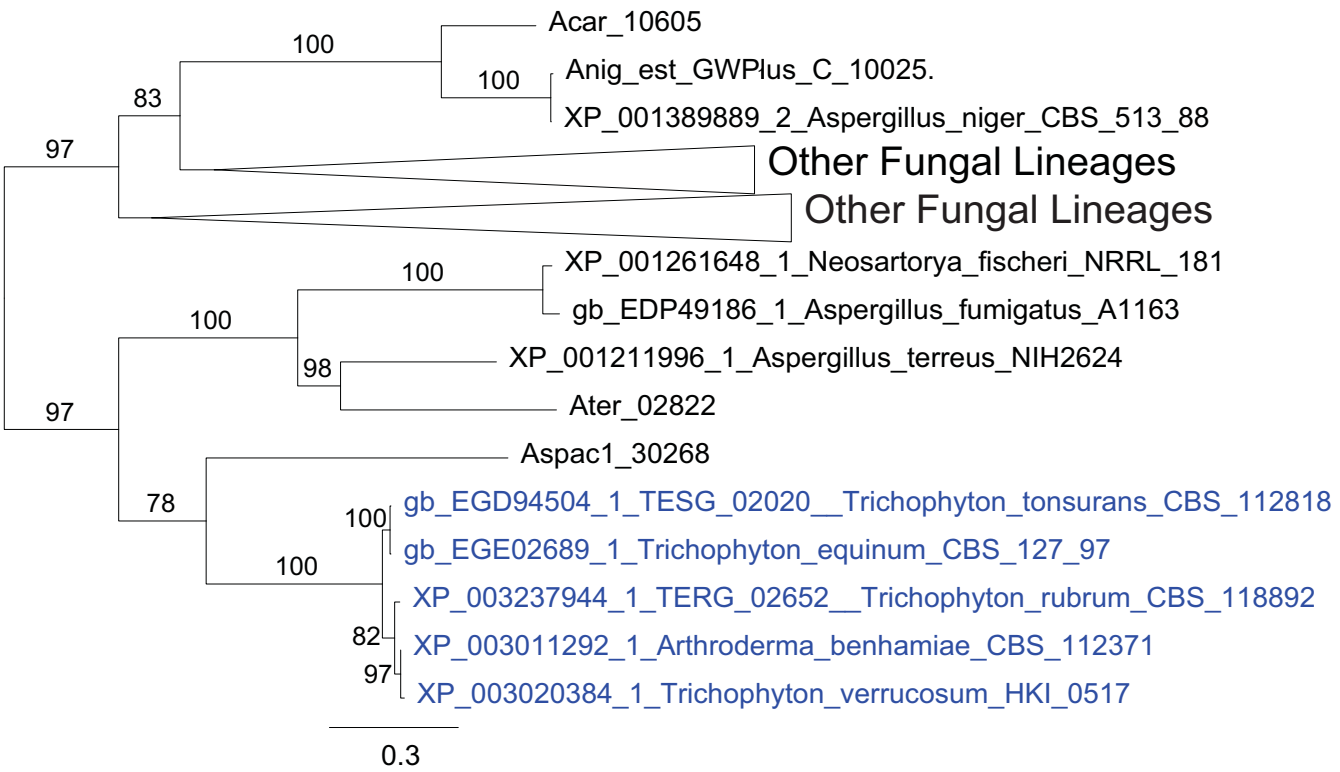

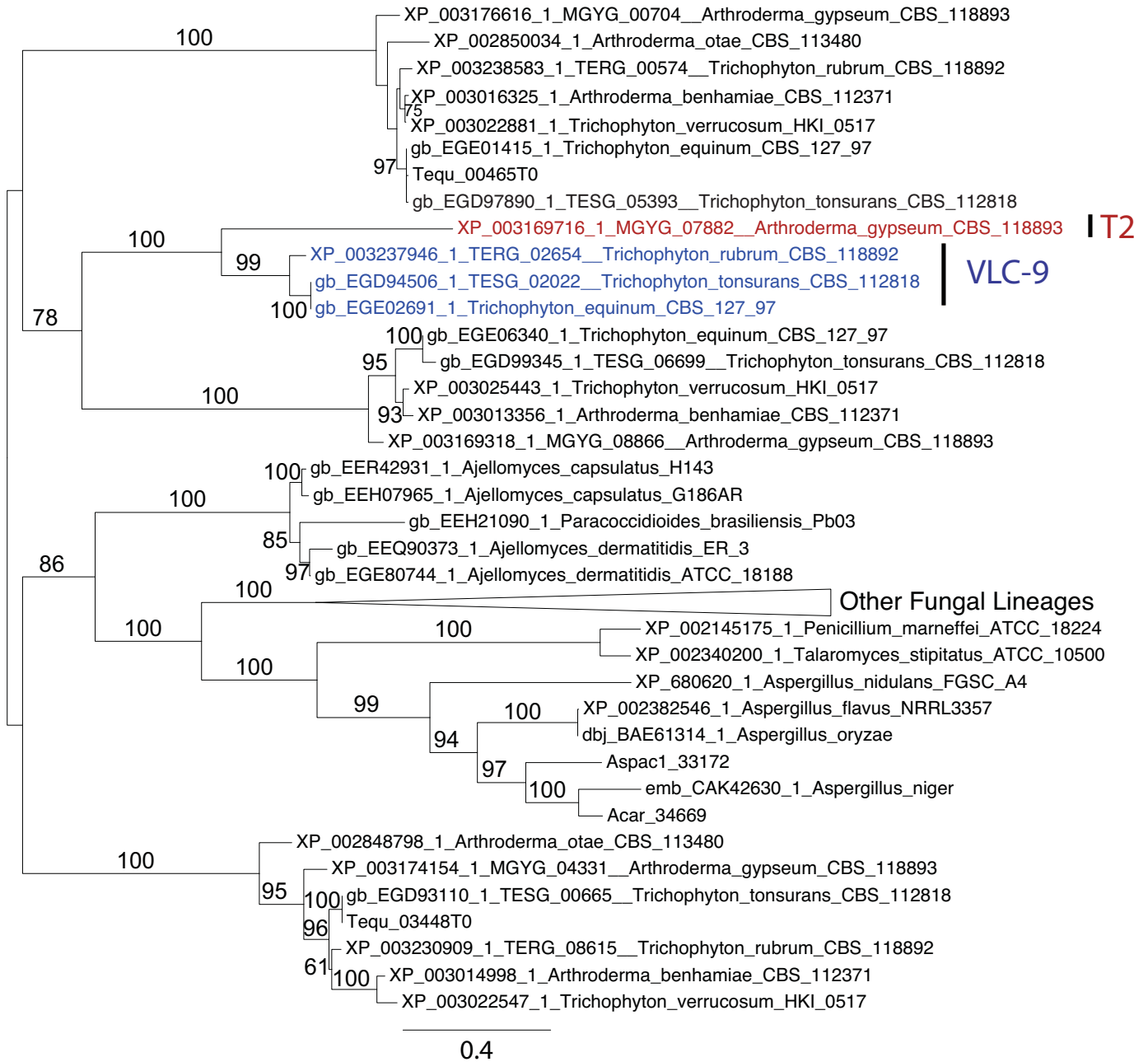

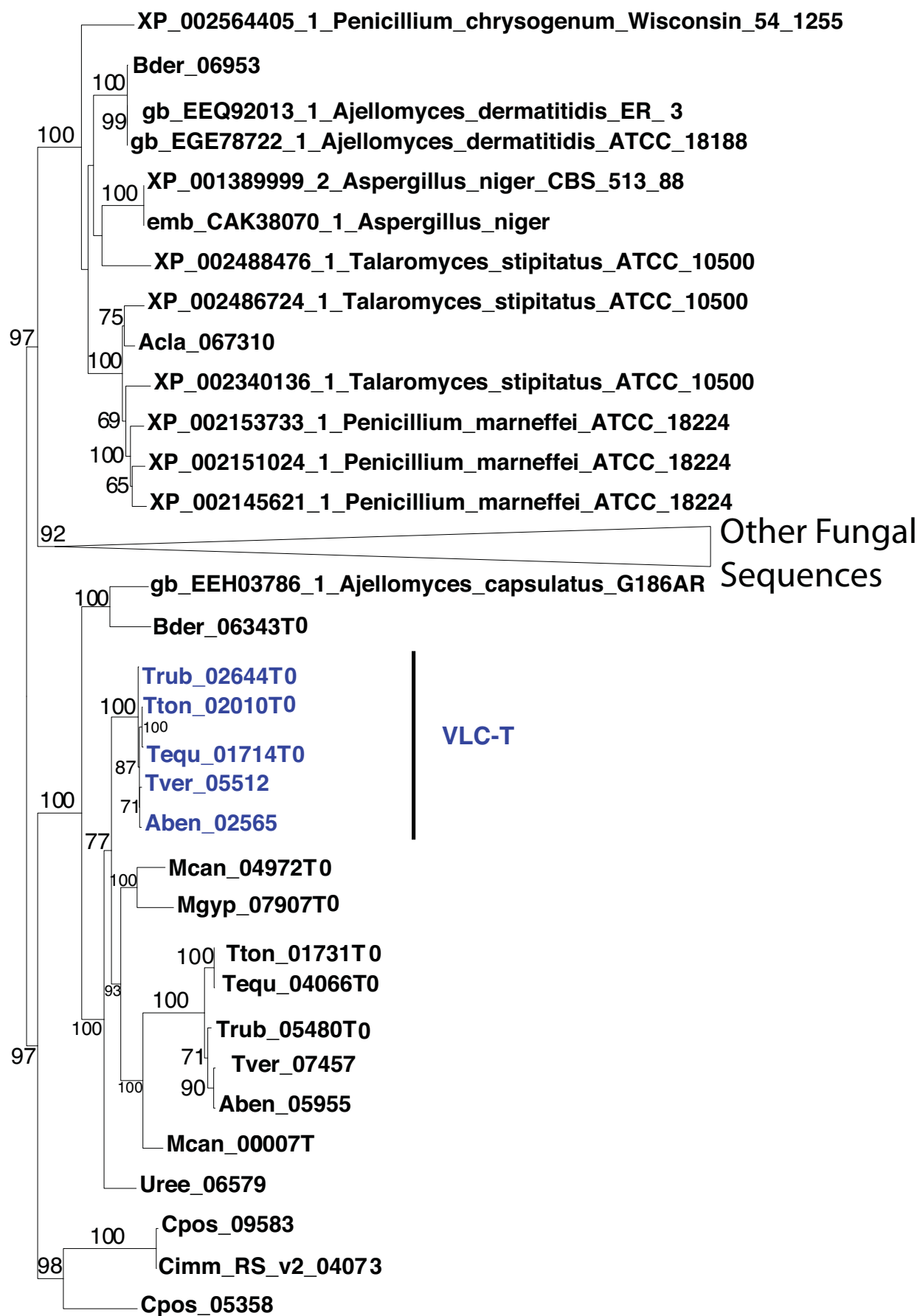

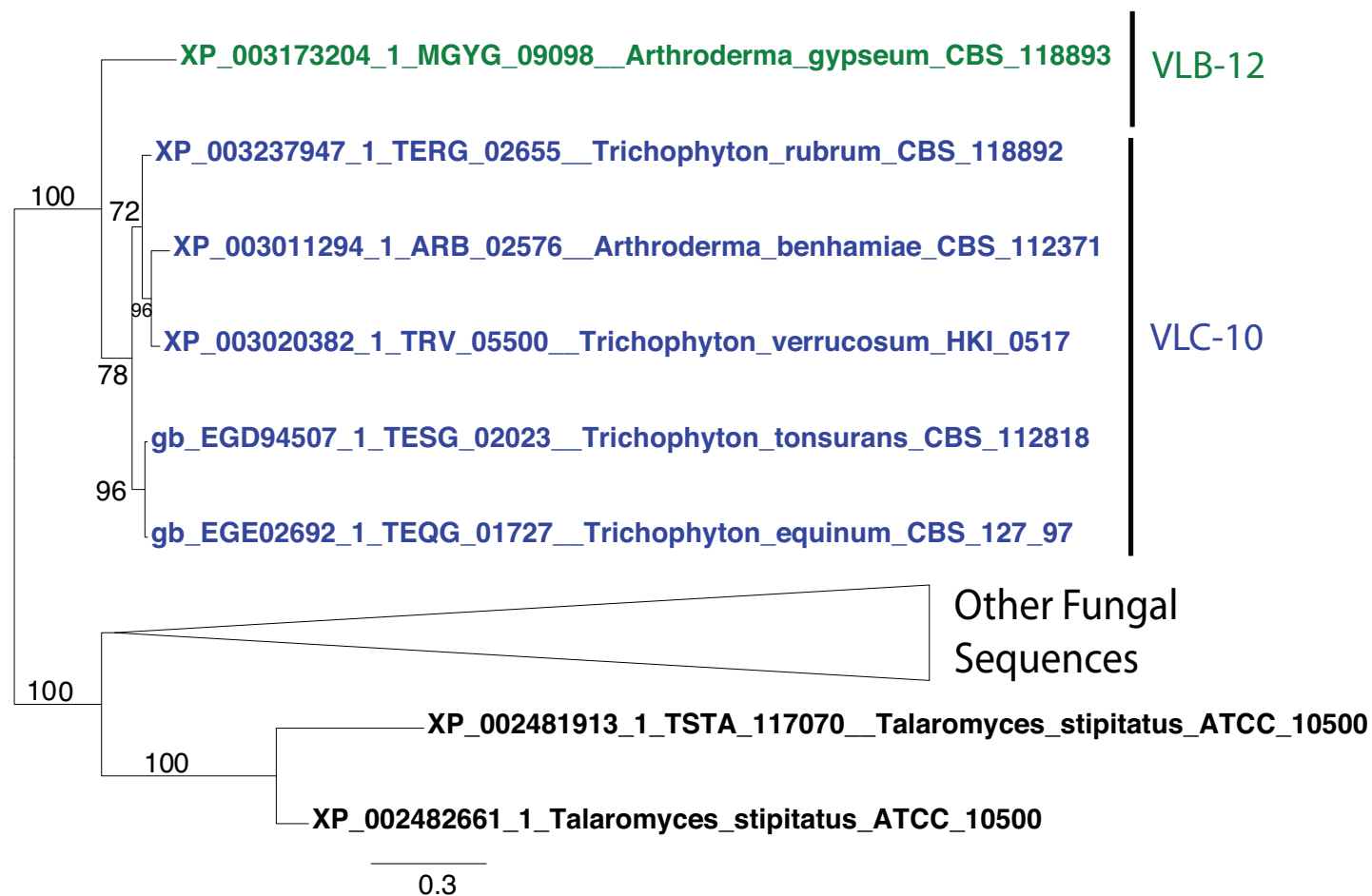

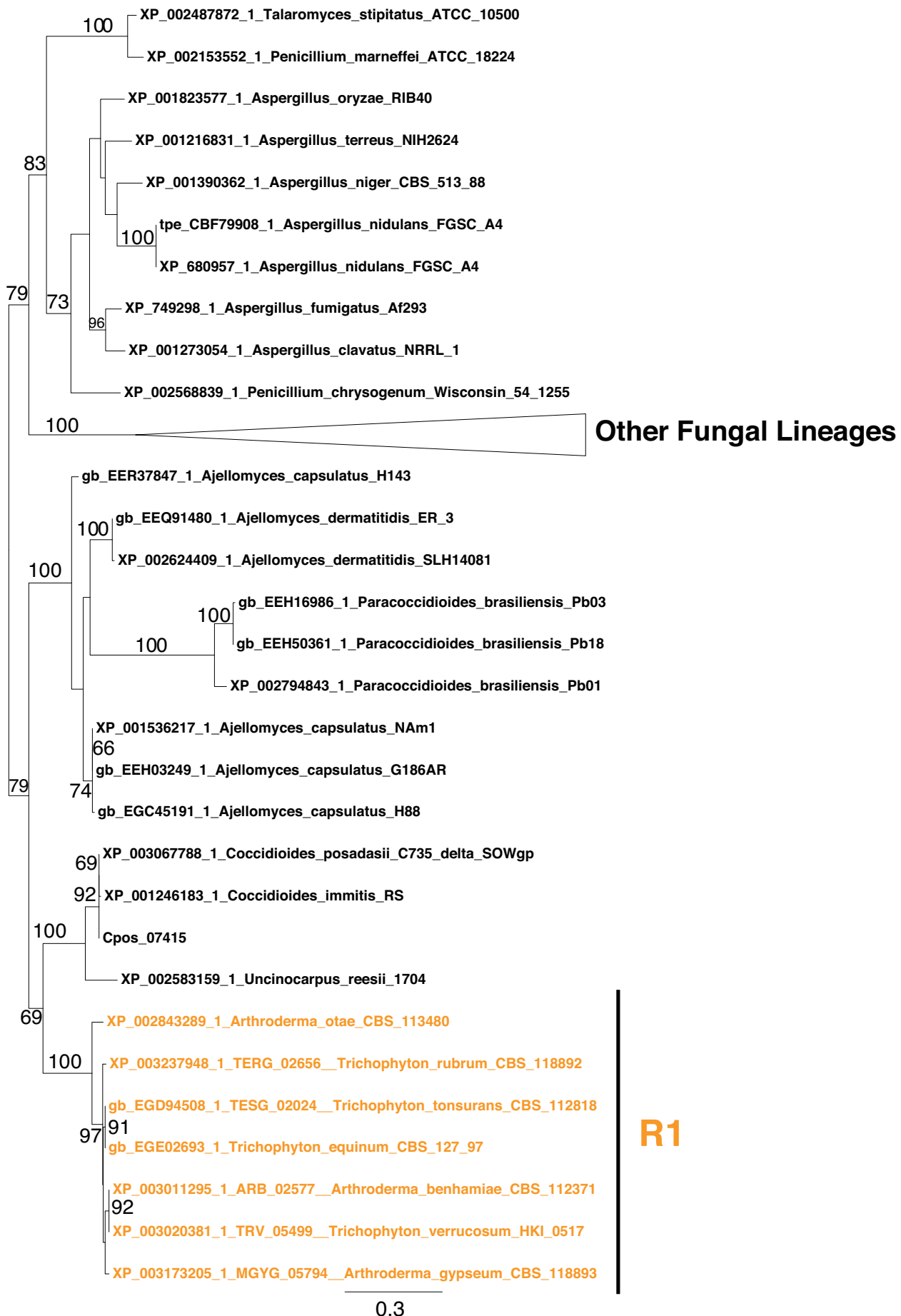

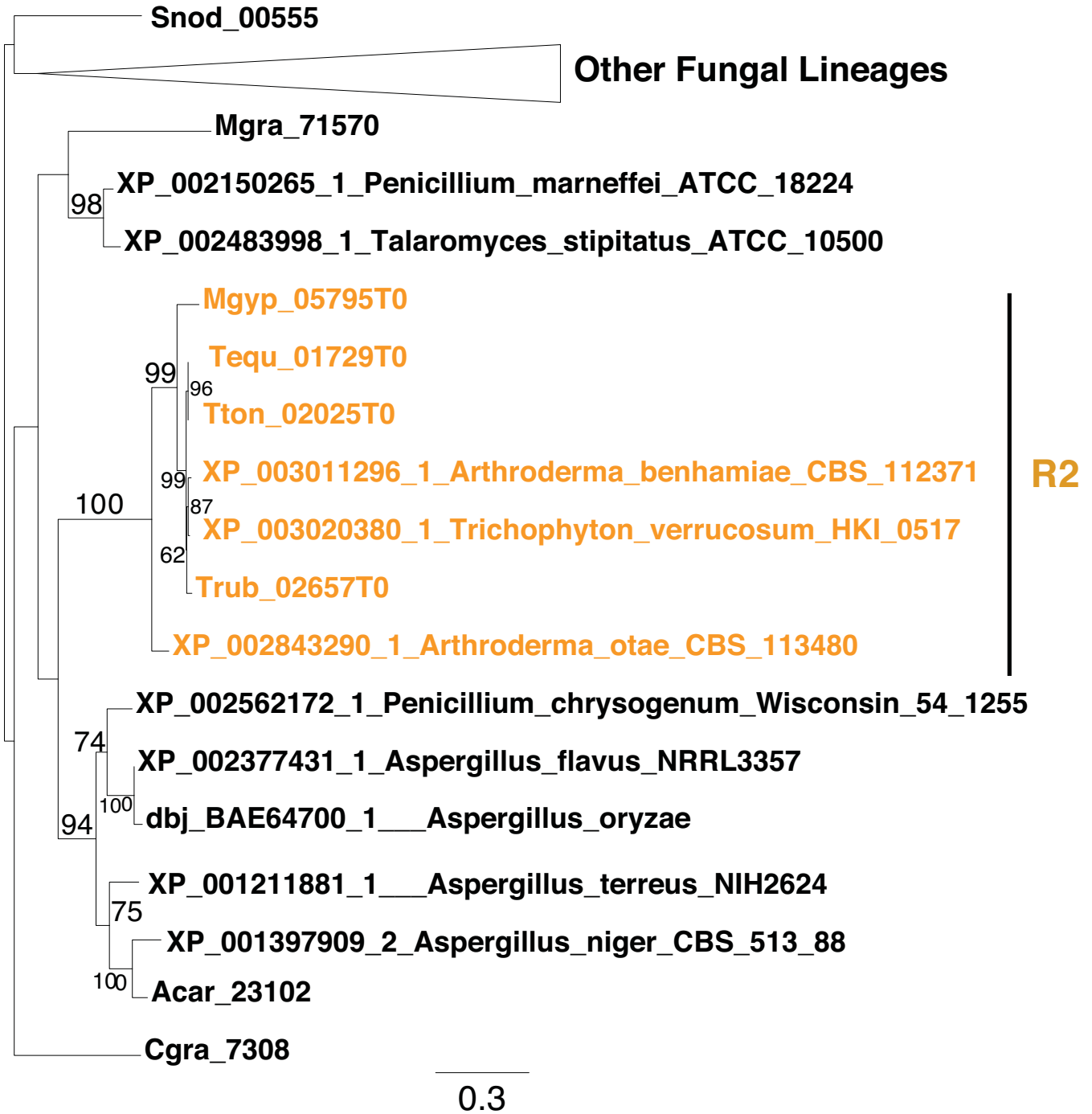

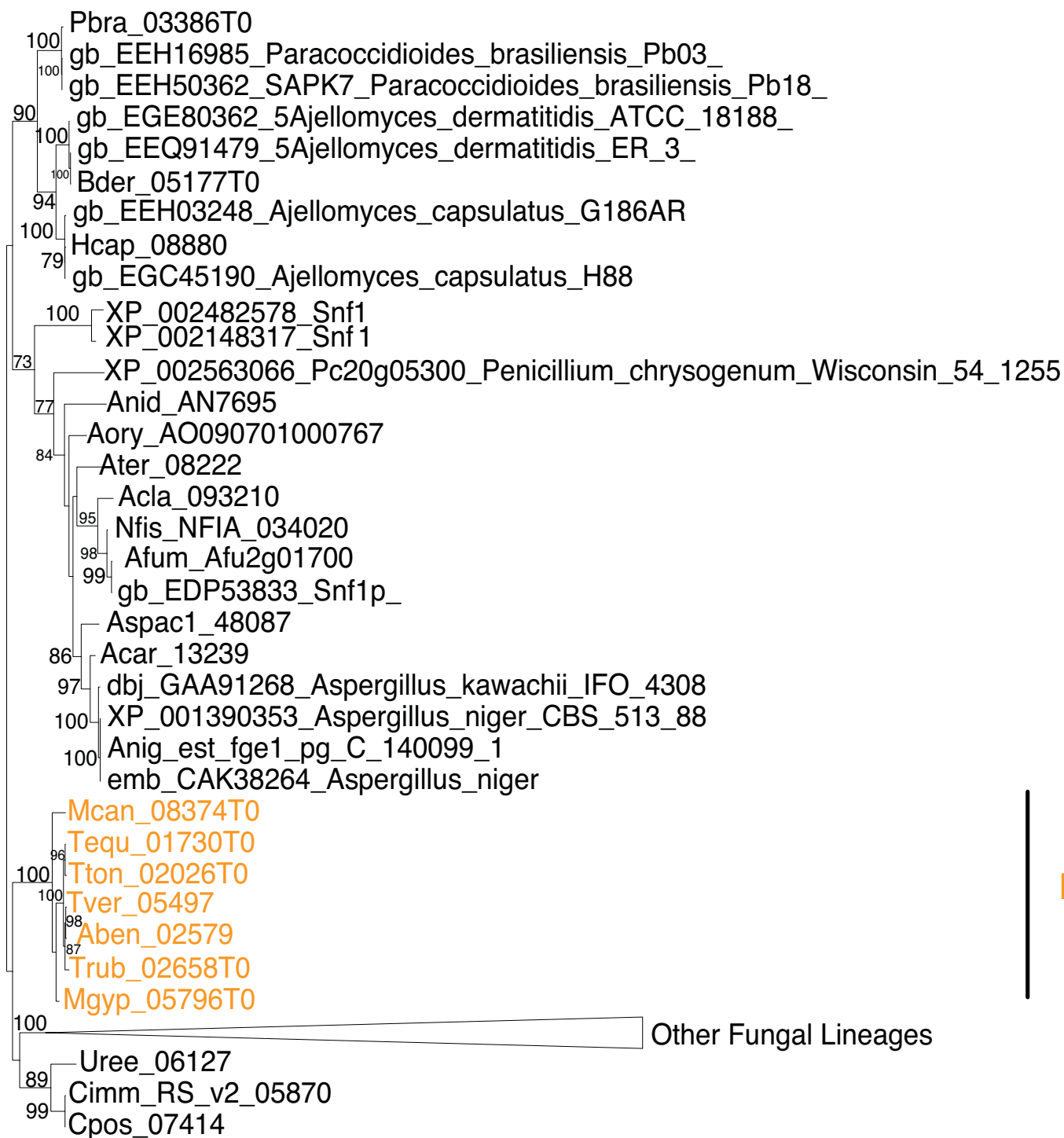

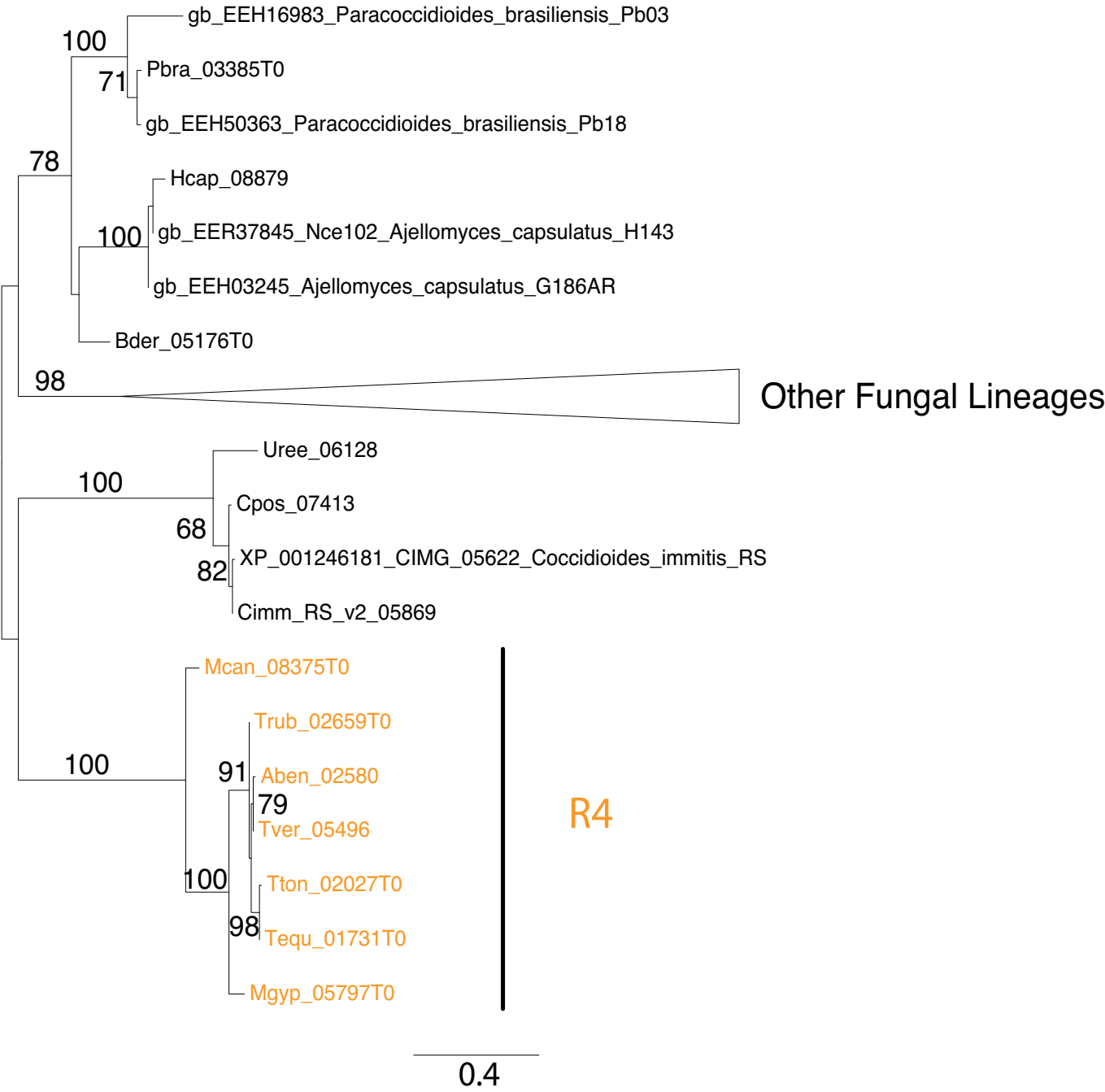

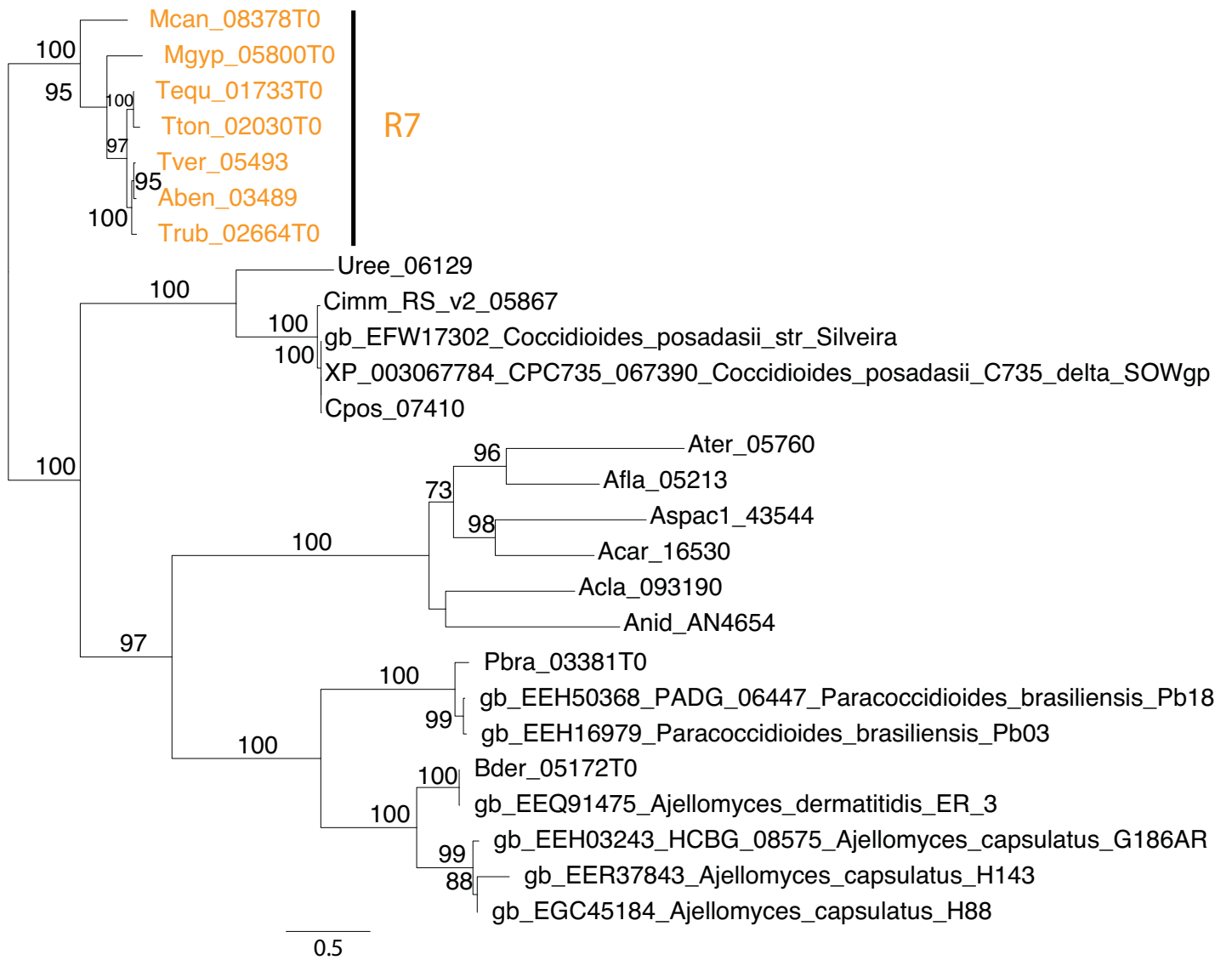

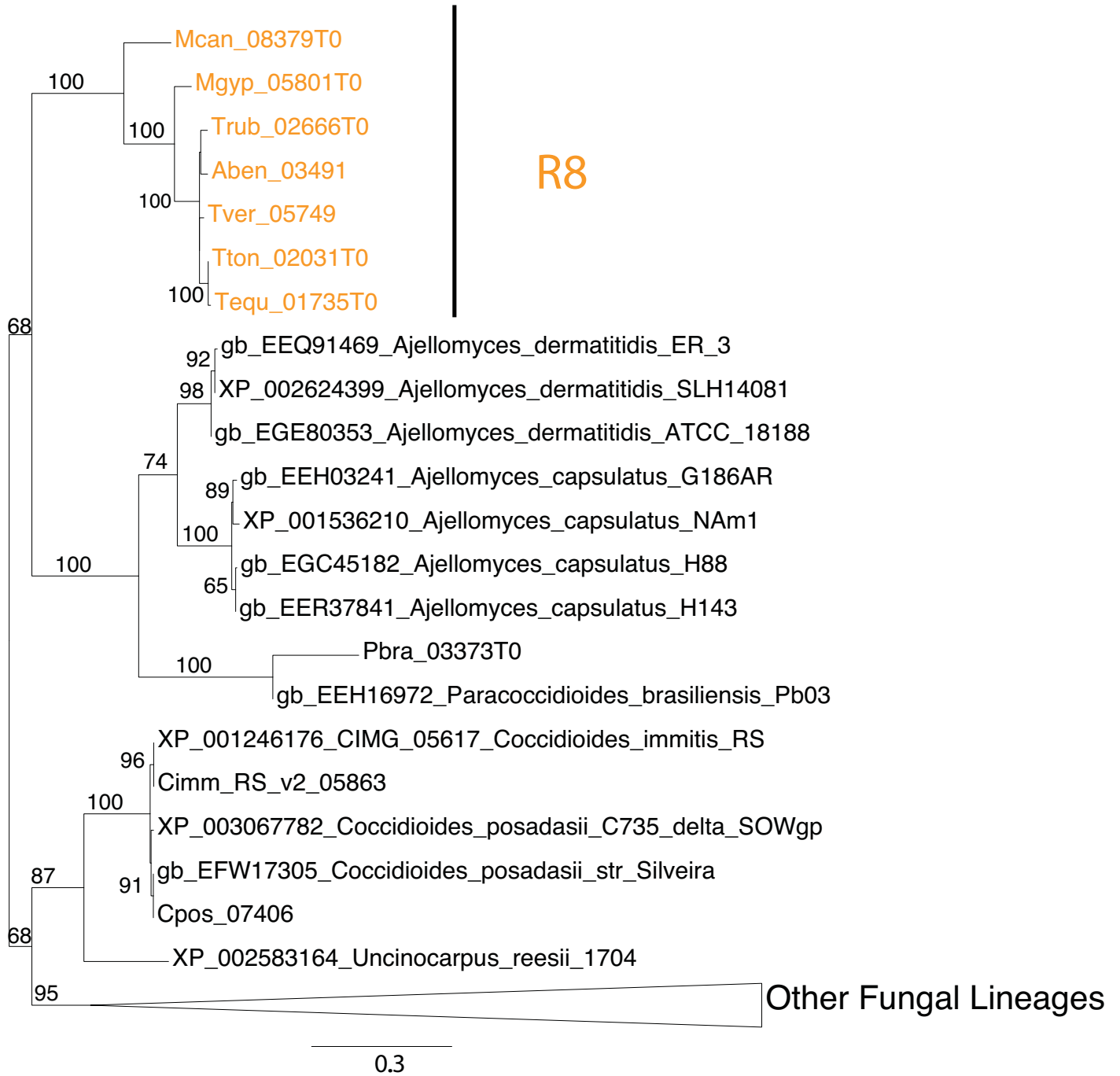

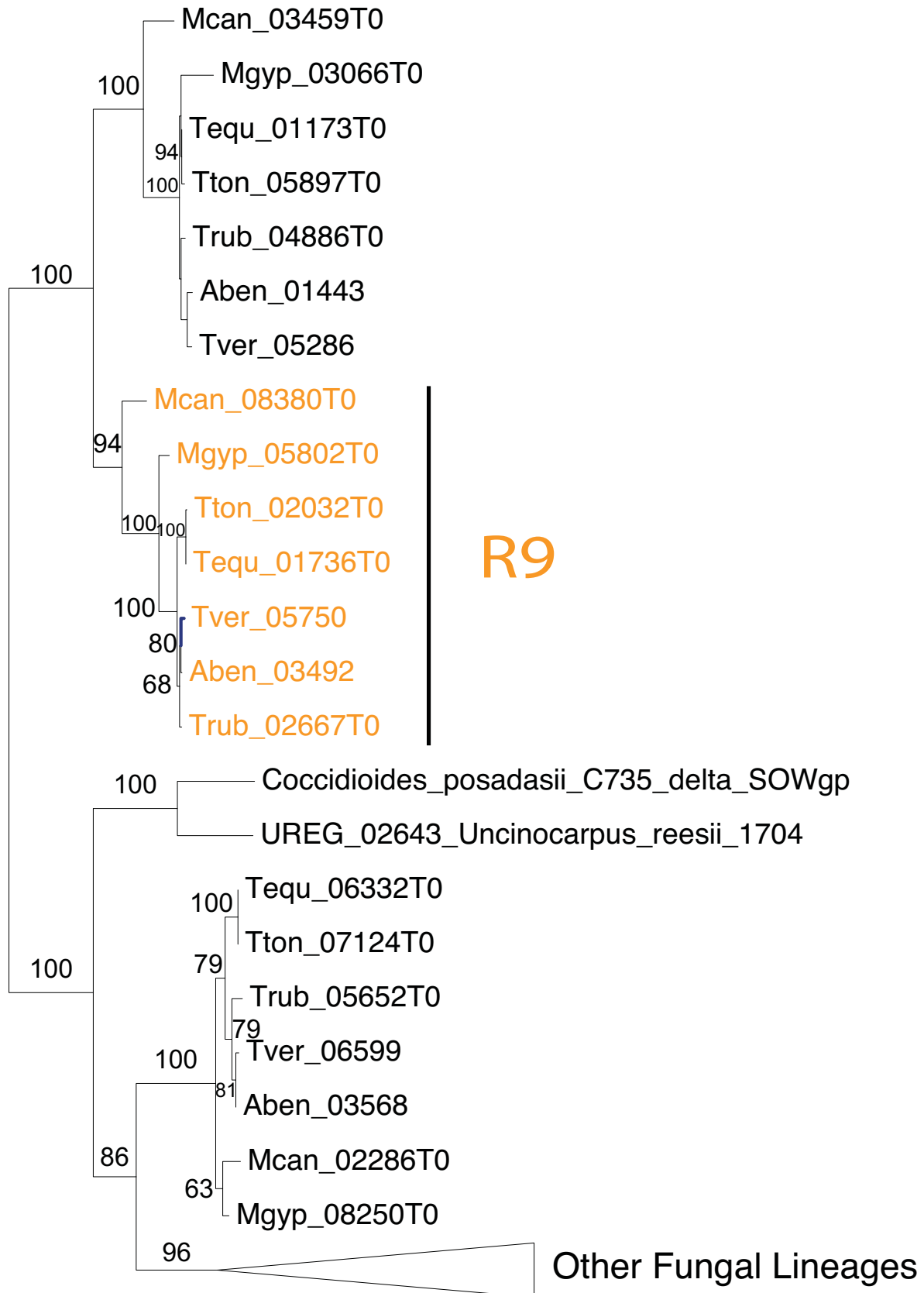

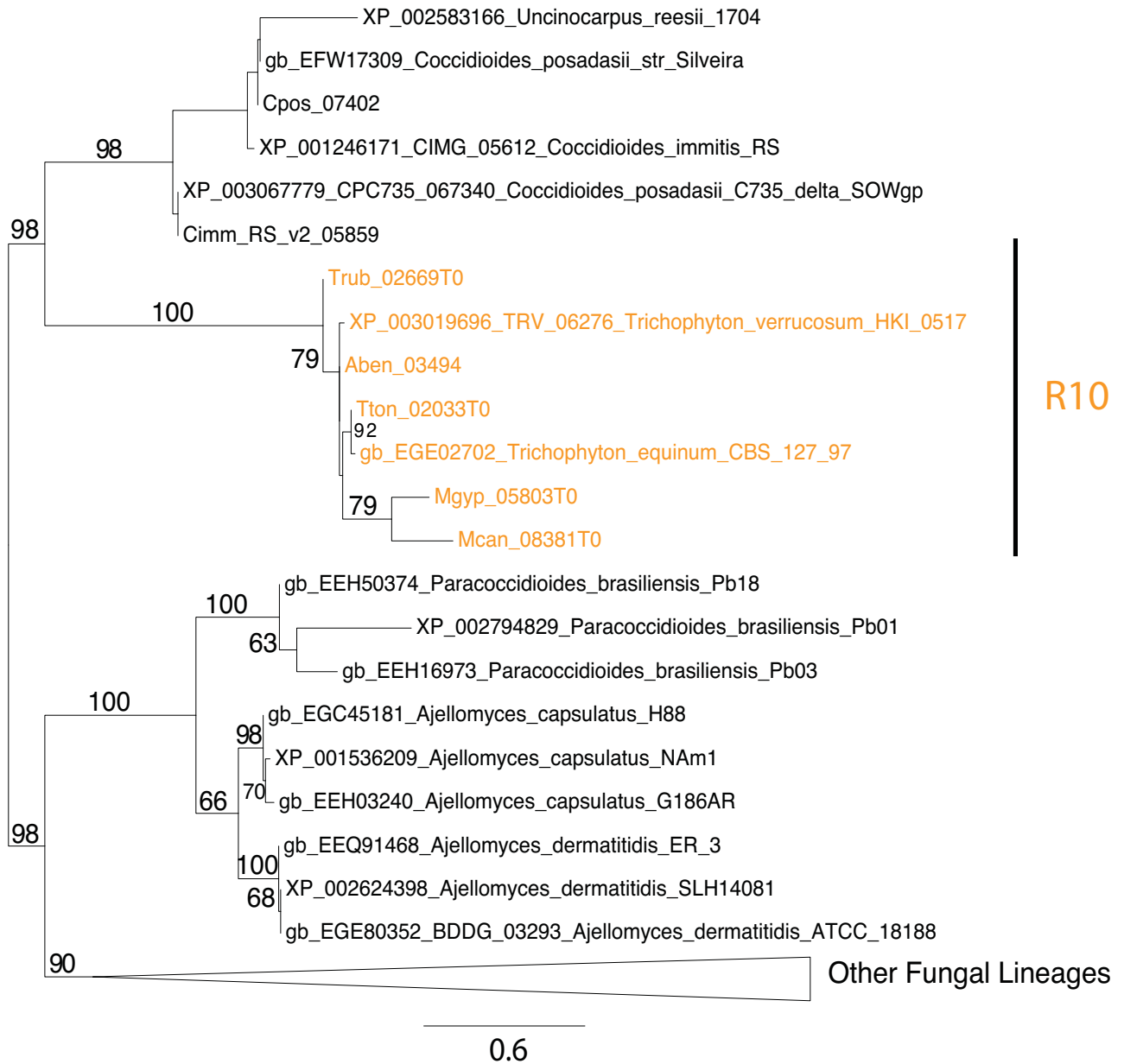

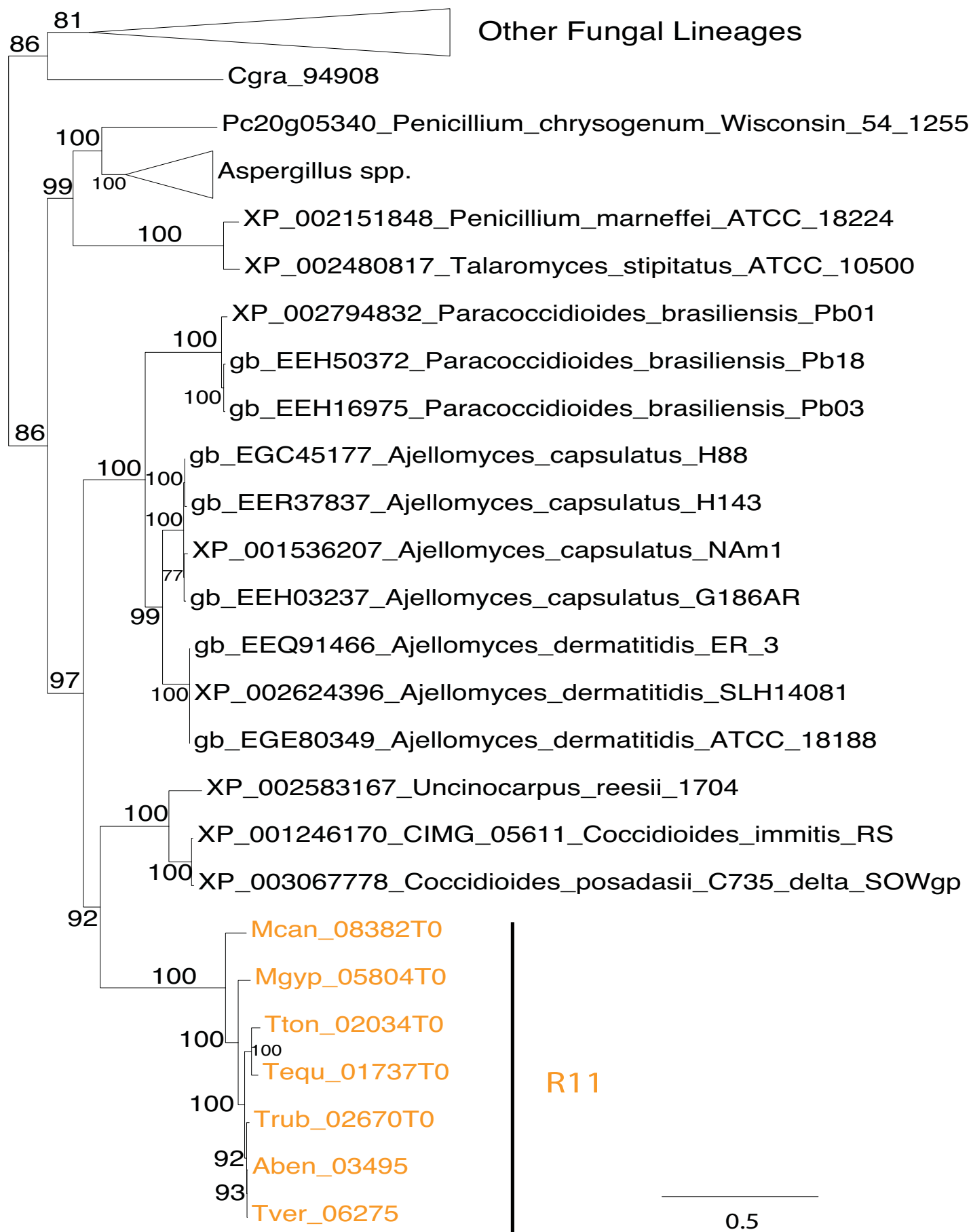

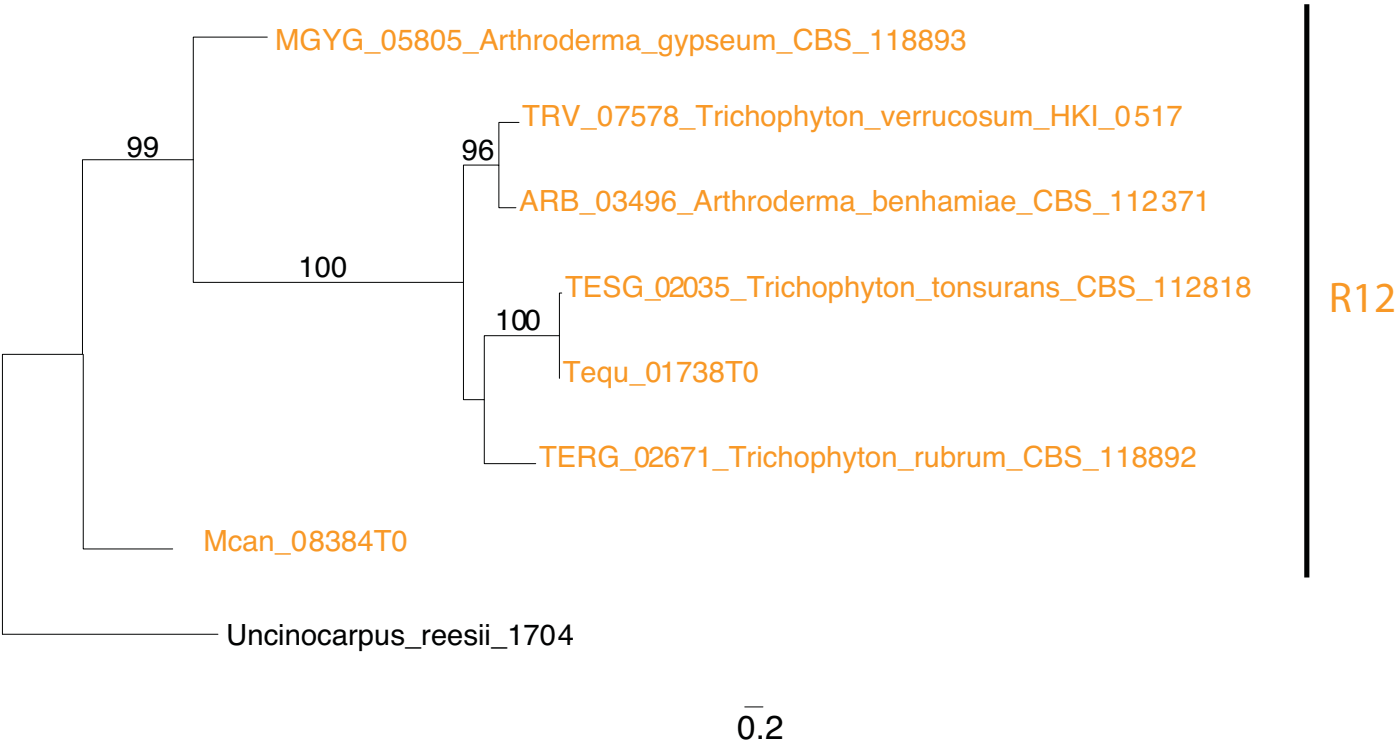

Supplement: Figure S2 — Phylogenetic trees of proteins encoded by the VL and flanking regions. (A–F) Highlighted sequence names labeled with “L1–6” are orthologs of genes on the 5′ flank of the VL, and (AA–AJ) highlighted sequence names labeled with “R1–12” are orthologs of genes on the 3′ flank of the VL. R5 and R6 are genes predicted in M. gypseum that were excluded from the analysis due to absence of orthologous sequences from other genomes. (G–Z) VLB and VLC gene orthologs are highlighted in green and blue respectively. Maximum Likelihood support values for nodes greater than or equal to 60% are shown. (PDF) [file pone.0041903.s002.pdf]

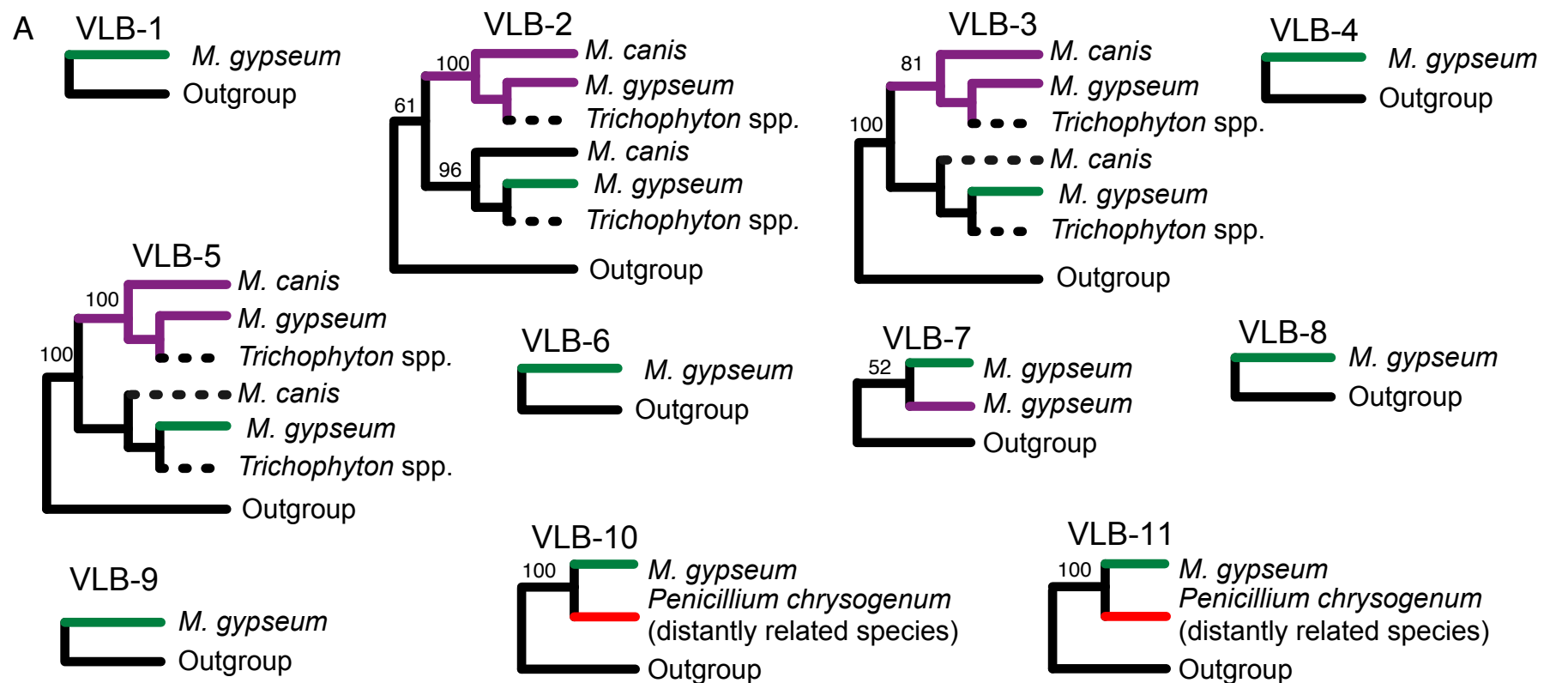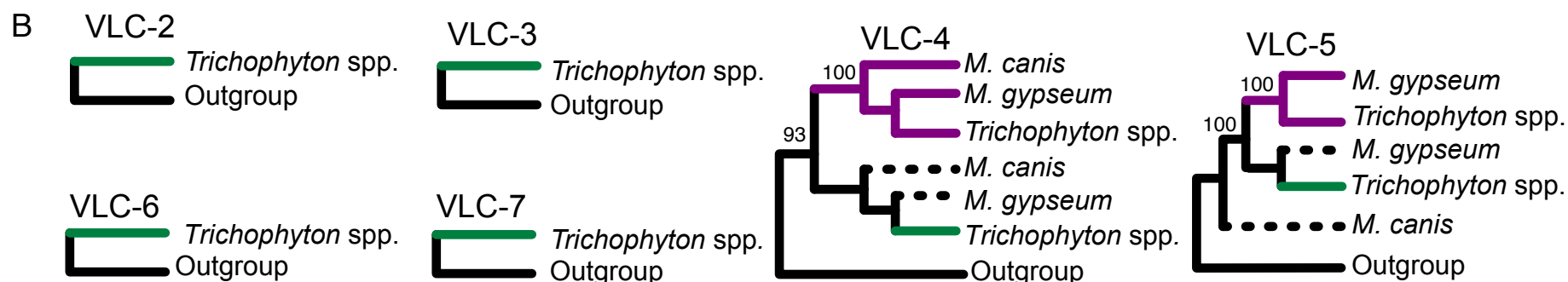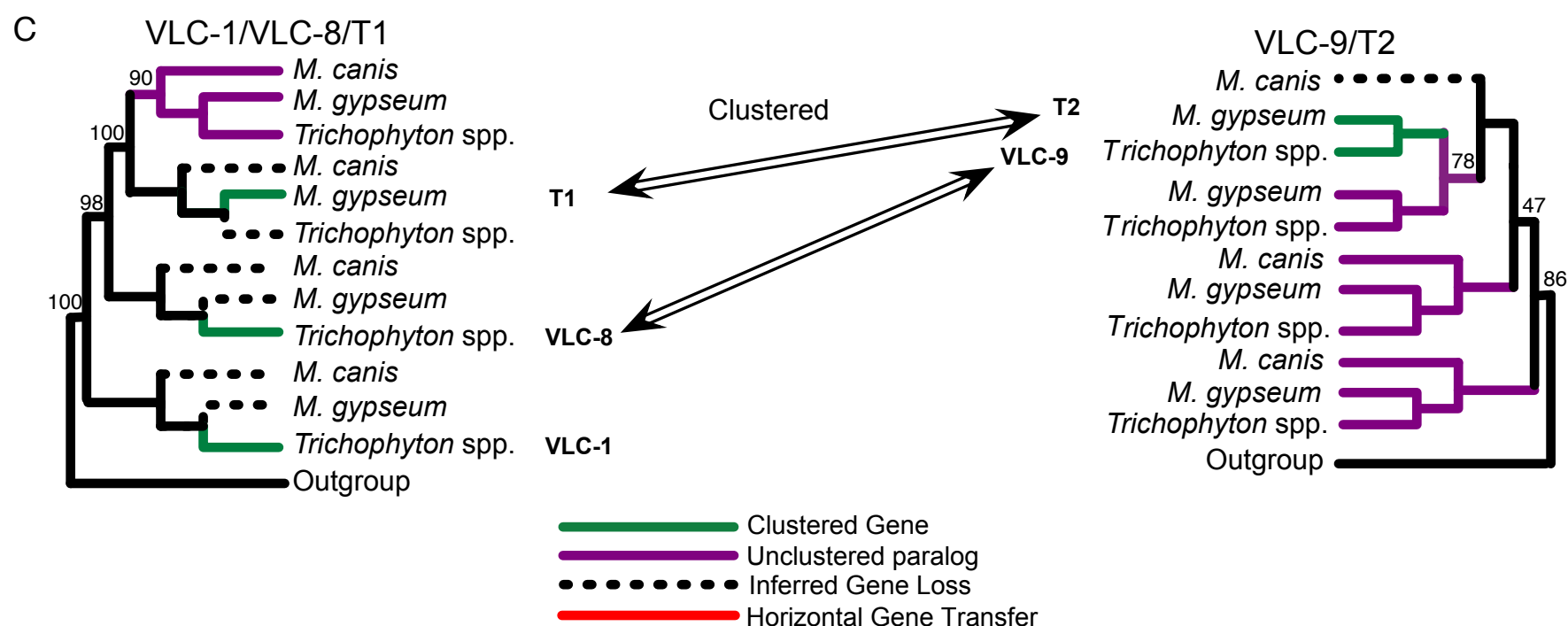

Supplement: Figure S3 — Parsimonious reconstructions of all VLB and VLC gene families. (A) Reconstructions of gene duplication and loss in VLB gene families. (B) Reconstructions of gene duplication and loss in VLC gene families. (C) Reconstructions of gene duplication and loss in multigene families containing more than one paralog in the VL. Genes VLC-8, VLC-1, and T1 are in the same gene family and are displayed on the left panel. Genes T2 and VLC-9 are in the same gene family and are on the right panel. VLC-8 and VLC-9 are physically clustered in Trichophyton spp. genomes, whereas their paralogs T1 and T2 are physically clustered in the M. gypseum genome in another locus outside the VL. Maximum likelihood bootstrap support values greater than 60% are shown. Green branches represent sequences clustered in the VL, purple branches represent unclustered paralog sequences used to infer the ancestral status of genes, dotted branches represent sequences inferred to have been lost, and red branches represent sequences inferred to have been acquired via horizontal gene transfer. (PDF) [file pone.0041903.s003.pdf]
