# Supplementary material for: Two Different Secondary Metabolism Gene Clusters Occupied the Same Ancestral Locus in Fungal Dermatophytes of the Arthrodermataceae
Source: PLoS One. 2012 Jul 30;7(7):e41903. doi: 10.1371/journal.pone.0041903 (PMC3408471; doi:10.1371/journal.pone.0041903)
Supplement: Table S1 — Complete and draft genomes analyzed in this study. (PDF) [file pone.0041903.s004.pdf]

Species	Strain	Genome Center[1]
**Acremonium alcalophilum**	v1.0	JGI
**Agaricus bisporus var. bisporus**	H97	JGI
**Agaricus bisporus var. burnettii**	JB 137-S8	JGI
**Allomyces macrogynus**	ATCC 38327	Broad
**Arthroderma benhamiae**	CBS 112371	Broad
**Aspergillus aculeatus**	ATCC16872 v1.1	JGI
**Aspergillus carbonarius**	ITEM 5010	JGI
**Aspergillus clavatus**	NRRL 1	TIGR/JCVI
**Aspergillus flavus**	NRRL 3357	TIGR/JCVI
**Aspergillus fumigatus**	Af293	TIGR/JCVI
**Aspergillus nidulans**	FGSC A4	Broad
**Aspergillus niger**	CBS518.33	DSM
**Aspergillus oryzae**	RIB40 / ATCC 42149	NITE
**Aspergillus terreus**	NIH 2624	Broad
**Auricularia delicata**	SS-5 v1.0	JGI
**Batrachochytrium dendrobatidis**	JEL423	Broad
**Blastomyces dermatitidis**	SLH-14081	Broad
**Botrytis cinerea**	B05.10	Broad
**Candida albicans**	SC5314	Biozentrum
**Candida glabrata**	CBS138	Genolevures
**Candida guilliermondii**	ATCC6260	Broad
**Candida lusitaniae**	ATCC42720	Broad
**Candida parapsilosis**	CDC317	Broad
**Candida tropicalis**	MYA3404	Broad
**Ceriporiopsis subvermispora**	B	JGI

**Chaetomium globosum**	CBS 148.51	Broad
**Cladonia grayi**		Duke
**Coccidioides immitis**	RS	Broad
**Cochliobolus heterostrophus**	C5	JGI
**Coniophora puteana**	v1.0	JGI
**Coprinopsis cinerea**	Okayama7#130	Broad
**Cryphonectria parasitica**	EP155	JGI
**Cryptococcus gattii**	R265	Broad
**Cryptococcus gattii**	WM276	CMSGSC
**Cryptococcus neoformans**	B-3501A	Stanford University
**Cryptococcus neoformans**	JEC21	TIGR/JCVI
**Cryptococcus neoformans var. grubii**	H99	Broad
**Dacryopinax sp.**	DJM 731 SSP1	JGI
**Debaryomyces hansenii**	CBS 767	Genolevures
**Dichomitus squalens**	v1.0	JGI
**Dothistroma septosporum**	NZE10 v1.0	JGI
**Encephalitozoon cuniculi**	GB-M1	Genoscope
**Eremothecium gossypii**	ATCC 10895	Stanford University
**Fomitiporia mediterranea**	v1.0	JGI
**Fomitopsis pinicola**	SS1 v1.0	JGI
**Fusarium graminearum**	NRRL 31084	Broad
**Fusarium oxysporum**	4287 (race 2, VCG 0030)=FGSC 4286=NRRL 34936	Broad
**Fusarium verticillioides**	7600=FRC M3125=NRRL 20956	Broad
**Gloeophyllum trabeum**	v1.0	JGI

**Hansenula polymorpha**	CBS4732	Institute for Microbiology, Heinrich-Heine University Düsseldorf
**Heterobasidion annosum**		JGI
**Histoplasma capsulatum**	NAm1 (WU24)	Broad
**Hysterium pulicare**		JGI
**Kluyveromyces lactis**	CLIB210	Genolevures
**Kluyveromyces thermotolerans**	CBS6340	Genolevures
**Kluyveromyces waltii**	NCYC2644	Broad
**Laccaria bicolor**	S238N-H82	JGI
**Leptosphaeria maculans**		JGI
**Lodderomyces elongisporus**	NRRL YB-4239	Broad
**Magnaporthe grisea**	70-15	Broad
**Magnaporthe oryzae**	70-15	Broad
**Magnaporthe poae**	ATCC 64411	Broad
**Malassezia globosa**	CBS 7966	JGI
**Melampsora laricis-populina**	98AG31	JGI
**Microsporum canis**	CBS 113480	Broad
**Microsporum gypseum**	CBS 118893	Broad
**Mucor circinelloides**	CBS 277.49	JGI
**Mycosphaerella fijiensis**	CIRAD86	JGI
**Mycosphaerella graminicola**	IPO323	JGI
**Nectria haematococca**	MPVI	JGI
**Neosartorya fischeri**	NRRL 181	TIGR/JCVI
**Neurospora crassa**	OR74A	Broad
**Neurospora discreta**	FGSC 8579	JGI

**Neurospora tetrasperma**	FGSC 2508	JGI
**Paracoccidioides brasiliensis**	Pb01	Broad
**Phanerochaete chrysosporium**	RP-78	JGI
**Phycomyces blakesleeanus**		JGI
**Pichia membranifaciens**	v1.0	JGI
**Pichia stipitis**	CBS 6054.05	JGI
**Pleurotus ostreatus**	PC15	JGI
**Pneumocystis carinii**		CCHMC
**Podospora anserina**	DSM 980	Genoscope
**Postia placenta**	Mad-698-R	JGI
**Puccinia graminis f. sp. tritici**	CRL 75-36-700-3	Broad
**Punctularia strigosozonata**	v1.0	JGI
**Pyrenophora teres**	f. teres	JGI
**Pyrenophora tritici-repentis**	Pt-1C-BFP	Broad
**Rhizopus oryzae**	RA 99-880	Broad
**Rhodotorula graminis**	strain WP1 v1.1	JGI
**Rhytidhysteron rufulum**		JGI
**Saccharomyces bayanus**	623-6C (WashU), MCYC623 (Broad)	WashU/Broad
**Saccharomyces castellii**	NRRL_Y-12630	WashU
**Saccharomyces cerevisiae**	S288c	Broad
**Saccharomyces kluyveri**	NRRL_Y-12651	WashU
**Saccharomyces kudriavzevii**	IFO1802	WashU
**Saccharomyces mikatae**	IFO1815 (both WashU and Broad)	WashU/Broad
**Saccharomyces paradoxus**	NRRL_Y-17217	Broad
**Schizosaccharomyces japonicus**	yFS275	Broad

**Schizosaccharomyces octosporus**	yfs286	Broad
**Schizosaccharomyces pombe**	972h-	Sanger
**Sclerotinia sclerotiorum**	1980' strain (ATCC18683)	Broad
**Septoria musiva**	SO2202 v1.0	JGI
**Serpula lacrymans**	S7.9	JGI
**Setosphaeria turcica**	Et28A v1.0	JGI
**Spizellomyces punctatus**	DAOM BR117	Broad
**Sporobolomyces roseus**	IAM 13481	JGI
**Sporotrichum thermophile**	v2.0	JGI
**Stagonospora nodorum**	SN15	Broad
**Stereum hirsutum**	FP-91666 SS1 v1.0	JGI
**Taphrina deformans(EST only)**		TBestDB
**Trametes versicolor**	v1.0	JGI
**Tremella mesenterica Fries**	DSM 1558	JGI
**Trichoderma atroviride**	IMI 206040	JGI
**Trichoderma reesei**	QM6a	JGI
**Trichoderma virens**	Gv29-8	JGI
**Trichophyton equinum**	CBS 127.97	Broad
**Trichophyton rubrum**	CBS 118892	Broad
**Trichophyton tonsurans**	CBS 112818	Broad
**Trichophyton verrucosum**	HKI 0517	Broad
**Uncinocarpus reesii**	1704	Broad
**Ustilago maydis**	521	Broad
**Vanderwaltozyma_polyspora**	DSM 70294	YGOB
**Verticillium albo-atrum**	VaMs.102	Broad
**Verticillium dahliae**	VdLs.17	Broad

**Wickerhamomyces anomalus**	v1.0	JGI
**Wolfiporia cocos**	MD-104 SS10 v1.0	JGI
**Yarrowia lipolytica**	CLIB122	Genolevures
**Zygosaccharomyces rouxii**	CBS732	Genolevures

[1] TIGR/JCVI = J. Craig Venter Institute, DSM = Royal DSM N.V., Broad = The Broad Institute, NITE = National Institute of Technology and Evaluation, Biozentrum = Biozentrum Universität Würzburg, Genolevures = Bioinformatics Center · Bordeaux, JGI = DOE Joint Genome Institute, CMSGSC = Canada's Michael Smith Genome Sciences Centre, Genoscope = French National Sequencing Center, Stanford University = Saccharomyces Genome Database, Stanford University Department of Genetics, CCHMC = University of Cincinnati College of Medicine_Division of Infectious Diseases, WASHU = The Genome Center at Washington University, TbestDB = Taxinomically Broad EST Database, YGOB = Yeast Gene Order Browser, Duke = Duke's Institue for Genome Sciences and Policy
